# Antimicrobial Resistance in Aquaculture Environments: Unravelling the Complexity and Connectivity of the Underlying Societal Drivers

**DOI:** 10.1021/acs.est.2c00799

**Published:** 2022-09-14

**Authors:** Kelly Thornber, Abul Bashar, Md. Salahuddin Ahmed, Ashley Bell, Jahcub Trew, Mahmudul Hasan, Neaz A. Hasan, Md. Mehedi Alam, Dominique L. Chaput, Mohammad Mahfujul Haque, Charles R. Tyler

**Affiliations:** † Biosciences, Geoffrey Pope Building, University of Exeter, Stocker Road, Exeter EX4 4QD, United Kingdom; ‡ Centre for Sustainable Aquaculture Futures, University of Exeter, Stocker Road, Exeter EX4 4QD, United Kingdom; § Department of Aquaculture, Bangladesh Agricultural University, Mymensingh 2202, Bangladesh; ∥ Brahmaputra Laboratory, Quality Feed Ltd, Mymensingh 2200, Bangladesh

**Keywords:** Antimicrobial resistance, environment, aquaculture, food production, LMIC, antibiotic, DPSIR, framework

## Abstract

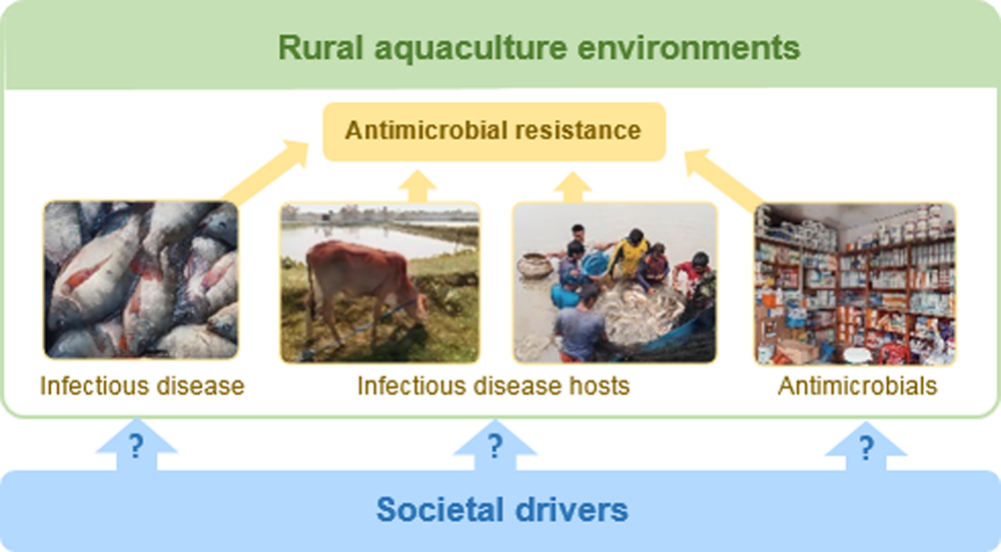

Food production environments in low- and middle-income countries (LMICs) are recognized as posing significant and increasing risks to antimicrobial resistance (AMR), one of the greatest threats to global public health and food security systems. In order to maximize and expedite action in mitigating AMR, the World Bank and AMR Global Leaders Group have recommended that AMR is integrated into wider sustainable development strategies. Thus, there is an urgent need for tools to support decision makers in unravelling the complex social and environmental factors driving AMR in LMIC food-producing environments and in demonstrating meaningful connectivity with other sustainable development issues. Here, we applied the Driver-Pressure-State-Impact-Response (DPSIR) conceptual framework to an aquaculture case study site in rural Bangladesh, through the analysis of distinct social, microbiological, and metagenomic data sets. We show how the DPSIR framework supports the integration of these diverse data sets, first to systematically characterize the complex network of societal drivers of AMR in these environments and second to delineate the connectivity between AMR and wider sustainable development issues. Our study illustrates the complexity and challenges of addressing AMR in rural aquaculture environments and supports efforts to implement global policy aimed at mitigating AMR in aquaculture and other rural LMIC food-producing environments.

## Abstract 2

This study provides a framework for decision makers to better understand and mitigate the risks of antimicrobial resistance in aquatic food-producing environments in low- and middle-income countries.

## Introduction

Antimicrobial resistance (AMR) is one of the greatest threats to global public health systems and food security, whereby the microbes that cause disease are becoming resistant to the drugs used to treat them. Food production environments are considered to pose a particularly high risk to the emergence and dissemination of AMR, especially in low- and middle-income countries (LMICs), where traditional extensive farming systems are moving toward more intensive practices.^1^ In many countries, this intensification is not accompanied by the infrastructural support needed to maintain good animal health and biosecurity, leading to rising levels of disease and an increased use of antimicrobial compounds (including antibiotics).^1^ Globally, more antibiotics are used in food production than in human healthcare, and usage in animals is expected to rise by 67% by 2030, predominantly due to this unsustainable intensification of food systems in LMICs.^1,2^ High levels of disease and antimicrobial usage are both key drivers of AMR, and so mitigating AMR risk in rural food producing environments in LMICs is a priority not only for global public health but also for food security and sustainable development programs more widely.^3^

Despite considerable motivation and momentum for addressing AMR at the highest international governance levels, global AMR levels are continuing to rise,^4^ exposing a significant “action gap”, with many countries struggling to translate political will into practice.^5−7^ Recent reports by the World Bank^7^ and the AMR Global Leaders Group^3^ suggest that the key to reducing this gap, especially in LMICs, is through integrating AMR into wider sustainable development policies; they call for major efforts in the field of AMR implementation research in order to increase our capacity to identify and characterize meaningful connectivity with wider sustainable development strategies. This would enable the measurement of potential co-benefits across a broad array of AMR-sensitive interventions in country-specific contexts, providing a more impactful and cost-effective approach to tackling AMR in LMIC contexts.^3,7^ In order to achieve this, decision makers first need to be able to understand the underlying drivers of AMR in a given context. This is particularly challenging for food production environments in rural LMICs, where AMR data are scant and disjointed, and it is recognized that a very wide range of socioeconomic factors are driving AMR emergence and transmission.^6^ These issues are not limited to LMIC food-producing environments, however, with studies showing that the environmental sector is poorly integrated in the AMR National Action Plans (NAPs) of many countries.^8,9^ Thus, tools are urgently needed to support decision makers in better understanding and mitigating AMR in complex environments.

**Figure 1:**
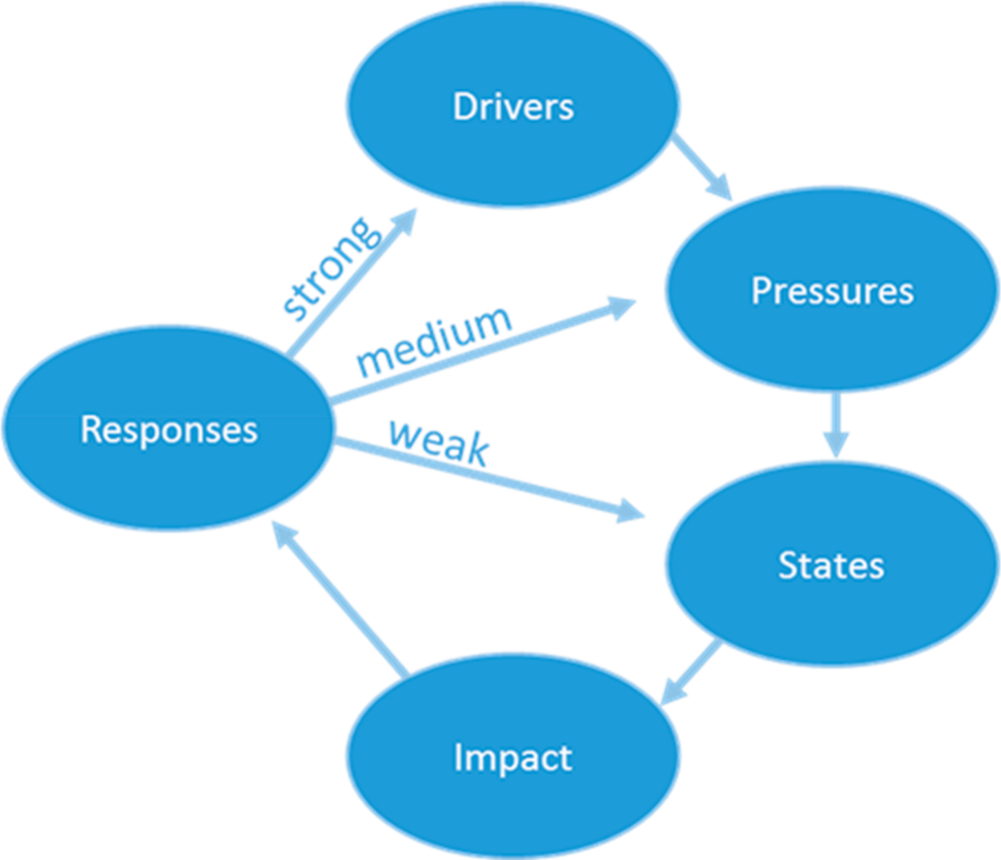
Driver-Pressure-State-Impact-Response (DPSIR) conceptual framework. We have adapted this framework to be able to systematically structure the issue of AMR in a rural LMIC aquaculture environment. Image is adapted from the European Environment Agency website.

Conceptual frameworks are tools widely used to bridge the gap between research outputs and the needs of decision makers for addressing complex environmental issues.^10^ DPSIR (Driver-Pressure-State-Impact-Response Framework)^11^ (Figure 1) is an established conceptual framework that has been used in low-, middle-, and high-income countries to better understand a variety of complex environmental sustainability issues in food production systems and has been proposed as a useful framework for improving AMR governance.^12^ In the original development of DPSIR,^11^ the “Drivers” were considered to be fundamental human, industrial, and societal needs, which drive human activities (the “Pressures”, e.g., production of waste and use of resources), that result in changes of “States” (e.g., water quality and population levels). Together, these lead to “Impacts” (e.g., on ecosystems and human health), which require societal and/or political “Responses”. Responses targeted further upstream of this causal pathway are considered to have the strongest impact (Figure 1). DPSIR provides a single framework for integrating social, cultural, and economic aspects of an issue and has been shown to allow the visualization of interactions within systems, identification of research gaps and intervention strategies, organization of information, and development of computational models.^13^

In this study, we explored whether the DPSIR framework could be applied to AMR in a complex food-producing environment case study, namely, that of a rural aquaculture environment in north-central Bangladesh, in order to understand and delineate the connectivity between AMR and wider sustainable development issues. Aquaculture is the fastest growing food sector globally, with industry expansion largely being facilitated through the rapid intensification of existing aquaculture farms in LMICs in Asia. Antibiotic usage in the industry is high and expected to rise with intensification and escalating disease levels,^14,15^ and a strong literature base now evidences that resistant microbes are present in aquaculture land and aquatic environments, as well as in animals within farm settings and at the point of sale.^15−18^ The international community recognizes the unique risks that aquaculture environments pose to global AMR;^19,20^ however, of the top five aquaculture-producing countries, only three of their existing AMR national action plans (NAPs) specifically mention aquaculture (Table S1). In the absence of effective aquaculture AMR management strategies, the AMR NAP plans that are in place remain predominantly focused on surveillance and monitoring rather than directly addressing the fundamental socioeconomic drivers of the issue.^21^ Here, we show how the DPSIR framework can be used to systematically identify the wider societal drivers of AMR in a rural aquaculture environmental context. Mapping the DPSIR outputs then allowed us to illustrate meaningful connectivity between AMR and broader sustainable development goals in these environments, providing a foundation for improving the implementation of national and global AMR policies.

## Materials and Methods

### Case Study

The Mymensingh region in north-central Bangladesh (Figure S1) was chosen as a case study for two main reasons: (i) We had three distinct AMR-related data sets available to us, allowing us to investigate AMR risk in this region. (ii) This region is considered typical of many other inland traditional aquaculture areas across Asia. Small-scale rural aquaculture contexts account for the majority of global aquaculture production, and these are the predominant farming systems in the Mymensingh region.^22^ Aquaculture has been a traditional way of life in Mymensingh for more than 40 years, and the region has become a major aquaculture production hub of Bangladesh due to favorable environmental and geophysical factors. Small scale aquaculture farms differ from the more commercial, intensive systems in that they are more closely interconnected within the local environment they share with humans and animals. Traditional rural aquaculture farms are typically a series of earthen ponds enclosed by dykes and land for crop/vegetable production. Rice and a variety of fish species are often cultivated either together (known as polyculture) or in succession, depending on seasonal and climatic conditions.^2^ Species include catfishes (predominantly Pangasius (*Pangasianodon hypophthalmus*)), Nile Tilapia (*Oreochromis niloticus*), various Indian and Chinese Carp species, perch, and barbs (a term referring to a number of ray-finned fish species). Farmers and their families live on site and often employ people from their local community. The region is now seeing increasing intensification of practices: in 2010–2011, the region produced 219,000 metric tonnes (MT) of fish, and by the 2018–2019 season, this had increased to 370,000 MT and accounted for 14.87% of national aquaculture production.^3,4^ There are thought to be around 112,000 aquaculture farmers in the region,^5^ with more than 11% of the population employed directly or indirectly through the aquaculture industry.^6^

### Metagenomic Data Set

In 2017–2018, pond water samples were collected from six different farms (see Table S2 for coordinates) on a monthly basis for microbial community profiling, as part of a UKRI-Newton Fund Global Research Partnership project investigating aquaculture pond microbiomes entitled *Prediction and Mitigation of Diseases Outbreaks in Aquaculture through Large Scale Community Engagement*.^23^ Triplicate water samples were collected from the same two or three ponds on each farm site every month over a period of one year. Sample collection involved passing pond water through a polycarbonate filter (47 mm diameter, 0.4 um pore size, Whatman) to collect the microbial biomass. The sample volume was determined by the concentration of suspended particulates in the water, so the end point was 200 mL or when water would no longer pass through the filter, whichever came first. Filters were immediately placed in 100% ethanol and kept at ambient temperature until they arrived in the laboratory in the United Kingdom, where they were stored at −20 °C until processing. Prior to DNA extraction, the ethanol was removed by freeze-drying at −110 °C (ScanVac CoolSafe Pro), and filters were then stored at −80 °C. DNA was extracted using an in-house CTAB/EDTA/chloroform method adapted from Bramwell et al.^24^ and Lever et al.^25^ The full protocol is available at https://www.protocols.io/view/ctab-chloroform-dna-extraction-from-ethanol-preser-rm7vz3rjrgx1/v2.

To provide sufficient amounts of DNA for PCR-free shotgun metagenomic sequencing, four seasonal time points were chosen, each consisting of a two-month period: peak of the monsoon (July/August 2017), postmonsoon (October/November 2017), winter (January/February 2018), and premonsoon (April/May 2018). Replicate pond DNA extracts from each of the six farms and from each of these four time points were pooled, totalling 24 samples. Pooled samples were cleaned using the Genomic DNA Clean & Concentrator-10 kit (Zymo Research), quantified with the Qubit dsDNA BR kit (ThermoFisher Scientific), and submitted to the Exeter Sequencing Service for PCR-free whole metagenome sequencing on the NovaSeq 6000 with the S1 Reagent Kit (300 cycles). Raw sequence data were deposited in the European Nucleotide Archive under BioProject number PRJEB53918.

For resistome profiling, metagenomic reads from all 24 pondwater samples were processed identically, with scripts available at https://github.com/ash-bell/AMR-Bangladesh-Fish-Ponds. Using an established BBTools v38.79 pipeline,^26^ reads were quality trimmed, and adaptors and synthetic artifacts were removed, decontaminated, and error corrected. Read assembly was performed by metaSPAdes v3.15.3^27^ using four *k*-mer lengths (25, 55, 95, and 125). Resistance genes were identified using the Resistance Gene Identifier (RGI) v5.2.1^28^ with the additional flags–alignment_tool BLAST – exclude_nudge −clean −low_quaility −split_prodigal_job. Plots were constructed in R using the ggplot2 package.^29^

For taxonomic and pathogenic identification, raw reads were first processed using a combination of Trimmomatic version 0.40^30^ and Sickle version 1.33^31^ to remove adaptor sequences and reads with quality scores less than 30 and/or shorter than 60bp. These quality filtered reads were then taxonomically classified using Kraken 2 v2.1.2^32^ and species abundances reassessed with Bracken 2.6.2.^33^ Pathogenic species were identified from the Kraken/Bracken output using the American Biological Safety Association (ABSA) database, accessed March 2021.^34^ To validate the identification of the most abundant pathogens from the Kraken 2 output, reads from each sampling site were mapped to the relevant reference genomes of the 10 most abundant (in terms of numbers of reads) pathogens identified for that sample. Alignments that had less than 50% coverage were not considered for further inferences on pathogen presence.

### Microbiological Data Set

Since October 2019, the Brahmaputra Lab of Quality Feed Limited, a pharmaceutical company based in Mymensingh, has been offering disease diagnostic services to farmers to identify the causative pathogen of disease in order to recommend and sell the most appropriate treatment. In their endeavors to help ensure that their antibiotic treatments are effective, they have been testing bacterial pathogens for resistance to a number of different antibiotics and kindly shared these data with us.

In their work, moribund fish were transported from farms around the Mymensingh region to the laboratory in pond water. Specific location data are not provided here in order to maintain farmer anonymity. External lesions, such as signs of necrotic skin, gill, or other external tissues or ulcers, were swabbed. In case of asymptomatic conditions, fish were dissected, and different organs (kidney, liver, gill, and external surface of digestive tract) were examined macroscopically to identify any abnormality and swabbed if affected. Due to the company’s aim of providing a rapid diagnostic service, CLSI guidelines were not strictly adhered to. Swabs were streaked directly onto selective media agar plates (listed per genera in Table S3A) and incubated at 35 °C for 36 h. If clinical signs of disease were observed, then diagnosis assumptions were made, and swabs were streaked only on media associated with the causative pathogen (e.g., visible edema with protruded anus in *Pangasius* gave an assumed etiology of *Edwardsiellosis*, so swabs were streaked only onto EIM media). If no colonies grew, then additional media were tested. Bacterial genera of isolates were confirmed via morphological characteristics and a range of biochemical tests, including gram staining, catalase, oxidase, indole, motility, citrate, urease, gas production, Voges–Proskauer, and a number of sugar tests, following the standard protocols as described by Holt et al.^35^ A lack of access to reference strains limited efforts for quality control. Following genera identification, resistance of isolates was assessed using the disc diffusion method according to Hombach et al.^36^ with some modification. In brief, pure inoculum was inoculated on Mueller–Hinton agar plates (preparation included 25 mL of liquid agar for each 100 mm disc). Commercial paper discs of 6 mm impregnated with antibiotics were placed on agar plates, and the plates were incubated at 35 °C for 24 h. The diameters of the zone of inhibition were measured using a slide calliper and SIRScan automatic reader (Montpellier Cedex, France). Antibacterial susceptibility was defined according to CLSI guideline inhibition zone diameter values^37^ (Table S3B**)**. Testing for resistance to levofloxacin and neomycin did not begin until May 2020. All antibiotics tested were classified as either critically (∗∗) or highly (∗) important for human health, according to the World Health Organization’s List of Critically Important Antimicrobials for Human Medicine.^38^

### Social Data Set

Socioeconomic data were collected by interviewing 30 farmers from the Mymensingh region in March 2021 as part of a United Kingdom Government ODA-funded project *Bangladesh Safe and Sustainable Aquatic Food - Embedding One Health to Support Aquatic Food Production during Covid-19*. Questions were designed to gain a holistic perspective of the issue of AMR in aquaculture (Table S4). Ethical approval for the data collection was received from the University of Exeter’s Biosciences Ethics Committee on 5 March 2021 (Reference: eCLESBio000401). Farming practices among the farmers in the Mymensingh region are very similar in terms of drug and chemical usage,^39^ and 30 representative farms from the Trishal, Phulpur, and Muktagacha districts were selected for participation. All gave written consent.

From our team’s experience, some farmers do not know which of the drugs and chemicals they use are antibiotics. Thus, prior to the interviews, we visited 41 farm shops in the region to gain a comprehensive list of the antibiotics that were on sale. A photographic inventory of the antibiotics was prepared so that farmers could identify which compounds they used from this list. Similarly, since many of the farmers do not have formal disease diagnosis training, for questions referring to infectious disease diagnoses of cattle, we sought to confirm etiologies, where possible, by asking for diagnosis reports or prescriptions issued by competent authorities. If these were not available, we asked for photos and information on allied clinical signs and symptoms, then validated these through consultation with veterinarians at Bangladesh Agricultural University Veterinary Clinic.

### Applying the DPSIR Framework

We defined a rural aquaculture environment as encompassing all culture ponds, the land within the aquaculture farm area (upon which crops are often grown), and all human/other animal inhabitants within an individual farm environment. The “Impact” was then taken to be the presence of AMR in these environments, thus the “States” were defined as the environmental conditions that together provide the optimal conditions for the development of AMR, namely, (1) presence of infectious disease, (2) presence of antimicrobial compounds, and (3) opportunities for human, animal, or plant exposure to infectious agents. For the purposes of this study, we considered only environmental states which directly lead to AMR emergence and/or transmission and therefore did not include commensals (nonpathogenic microbes that live on or in organisms). These states are based upon the framework for AMR environmental risk assessment proposed by Berendonk et al.^40^ to minimize the emergence and spread of AMR in the environment and its transmission into the community and are further described in Table S5. The underlying “Pressures” and “Drivers” of each “State” were then considered in turn, using our three data sets, existing literature, and our team’s extensive experience of working in this field. These were systematically documented, as shown in Table 1. In our study, we were seeking to meaningfully connect AMR to wider sustainable development strategies, so the “Responses” were identified as existing strategies that are in place to address the underlying “Drivers” and “Pressures” (Table S10).

**Table 1:**
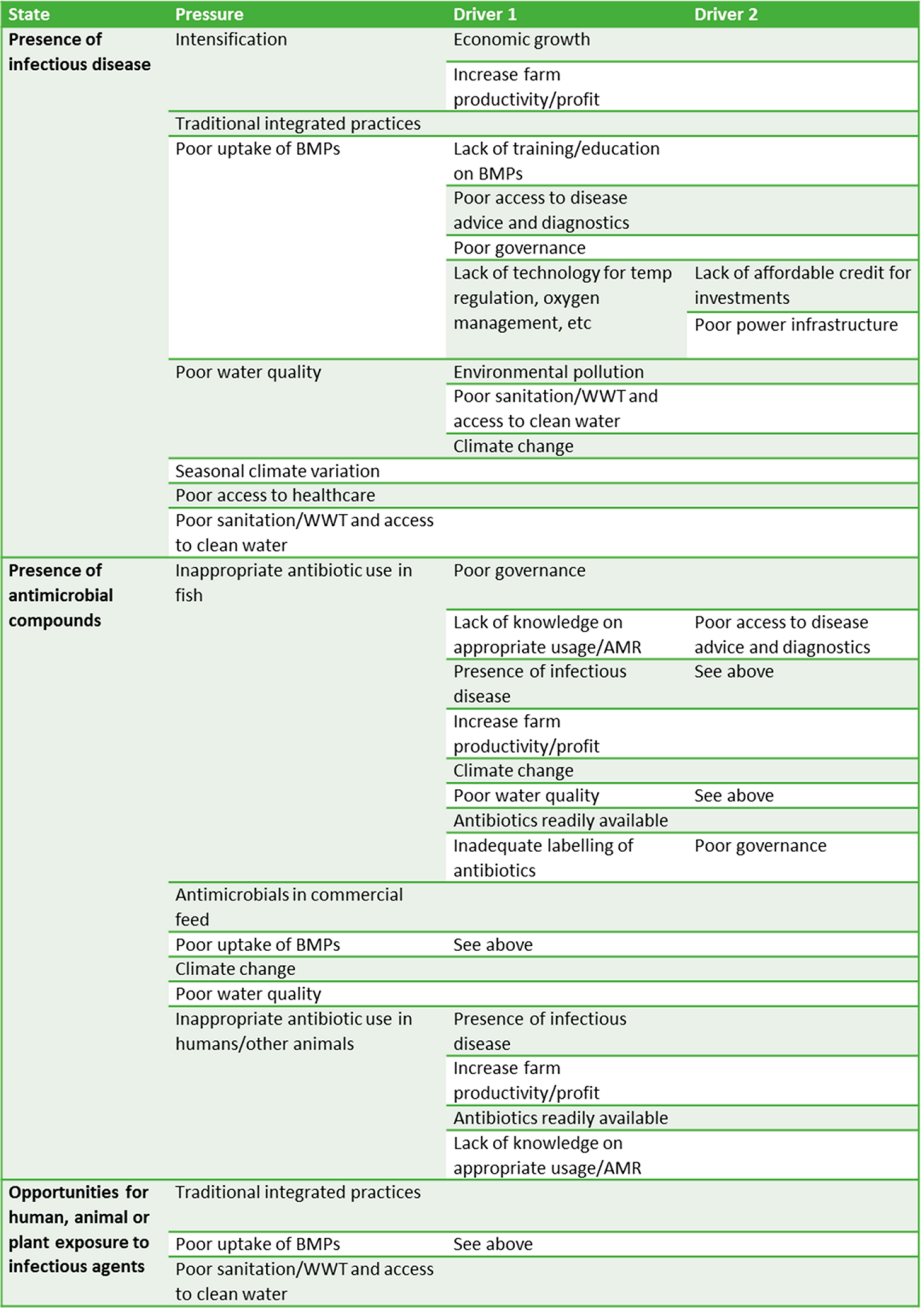
Documented DPSIR Results^a^

a BMPs: best management practices. WWT: wastewater treatment.

### Visualizing the DPSIR Outputs

To create the network map in Gephi version 0.9.2,^41^ the Drivers, Pressures, States, and Impact from Table 1 were listed in an attributes.csv file. Each individual connection identified in Table 1 was listed in the direction of Drivers → Pressures → States → Impact, with a weighting of 1, in an edgelist.csv file. These files (Table S6) were imported into Gephi and visualized using node-sized ranked according to out-degree.

## Results and Discussion

We applied the DPSIR framework to a case study of aquaculture environments in the Mymensingh region of north-central Bangladesh (Figure S1), for which we had access to three distinct data sets: (i) a microbiological data set, collected between October 2019 and September 2020 by Quality Feed Ltd., a feed and pharmaceutical company based within the Mymensingh region, (ii) a metagenomic DNA data set, in which water from six ponds was sampled and analyzed for four seasonal time points between April 2017 and February 2018, and (iii) a social data set, collected in February 2021 from 30 farmers that were representative of the wider aquaculture community in the region. More details of the case study region, the three data sets, and the application of the framework can be found in Materials and Methods.

### Evidencing the Impact: Presence of AMR

**Figure 2:**
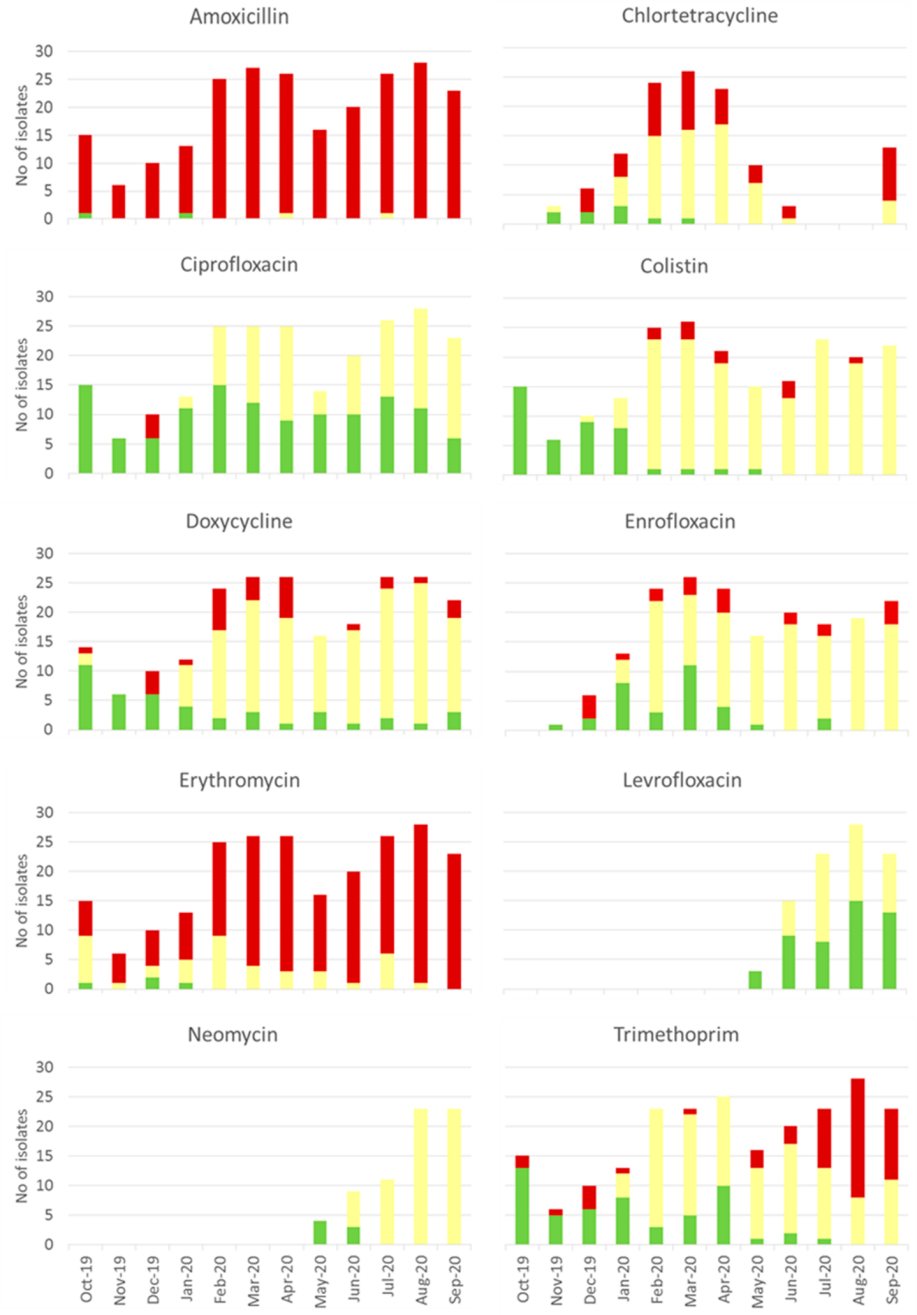
Microbiological data set showing resistance of bacteria isolated from diseased fish. Data collected by Quality Feed Ltd. Bacteria were isolated from diseased fish brought to the company’s laboratory for diagnosis between October 2019 and September 2020. Isolates were tested for resistance to these antibiotics, using the disc diffusion method, and classified as sensitive (green), intermediate susceptibility (yellow), or resistant (red), according to the criteria outlined in Materials and Methods. Bars show total number of isolates tested for each month. Testing for resistance to levofloxacin and neomycin did not begin until May 2020. All antibiotics tested were classified as either critically (∗∗) or highly (∗) important for human health, according to the World Health Organization’s List of Critically Important Antimicrobials for Human Medicine.^38^

We began by evidencing the “Impact”, which we defined as the presence of AMR in our case study aquaculture environment. Systematic AMR surveillance and monitoring data are not available to assess levels of AMR in the Mymensingh region, as is typical for the vast majority of aquaculture environments; however, all three of our data sets provided evidence of AMR. Our microbiological data set was limited to 12 bacterial genera isolated from diseased fish due to the laboratory methods and capacity at Quality Feed Ltd., but nevertheless clearly shows evidence of resistance, with very high levels of resistance in particular to amoxicillin and erythromycin (Figure 2, Tables S7 and S8). Some of the amoxicillin resistance may be attributed to the intrinsic resistance commonly reported in *Aeromonas* bacteria, which made up 34% (80 of 235) of the total isolates; however, amoxicillin resistance was also detected in almost all of the other bacterial genera isolated (Table S8).

**Figure 3:**
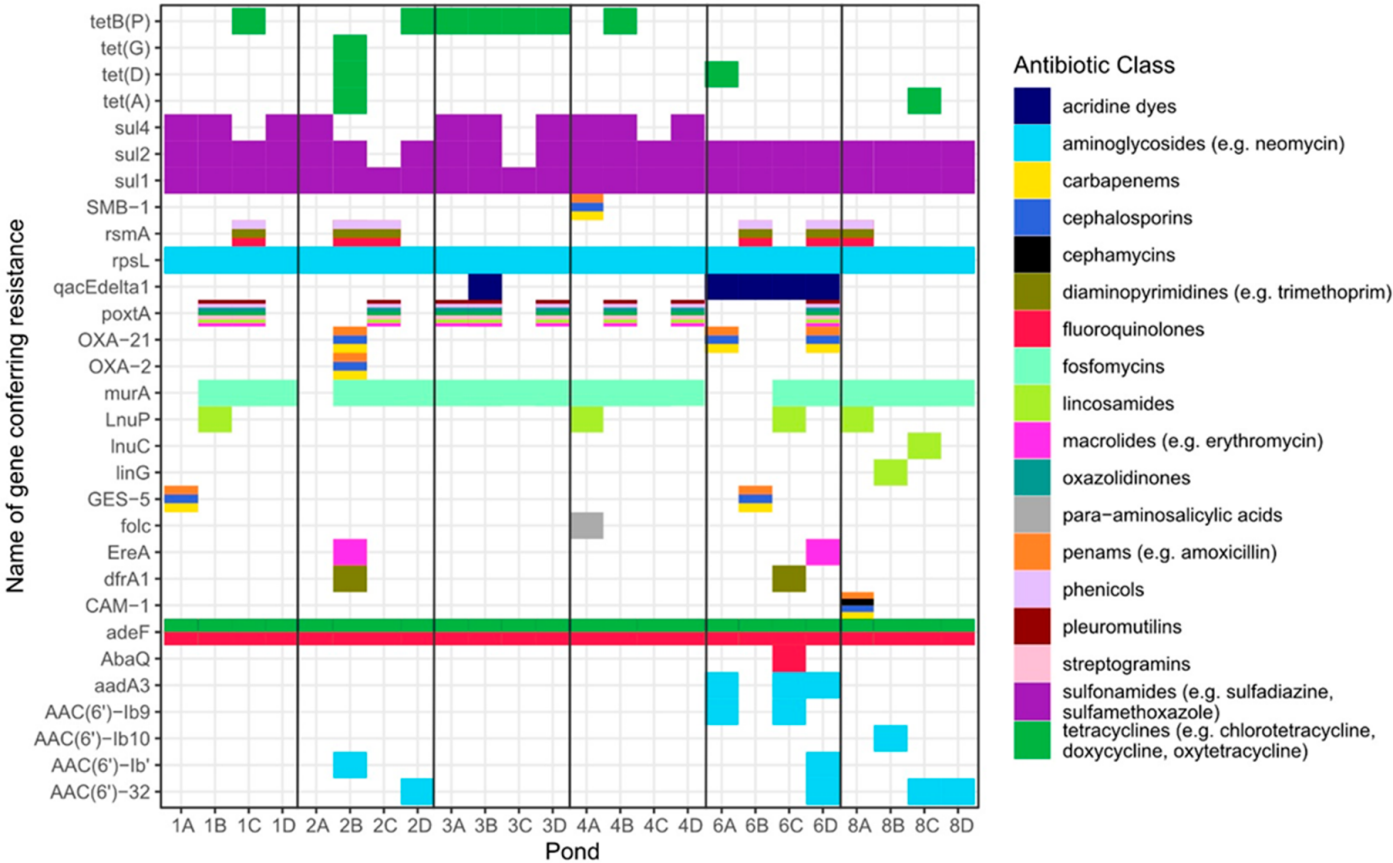
Metagenomic data set showing AMR genes detected in fish pond water samples. Between April 2017 and February 2018, pond water samples were collected from ponds at six farms, at four seasonal time points: (A) monsoon (Jul/Aug), (B) postmonsoon (Oct/Nov), (C) winter (Jan/Feb), and (D) premonsoon (Apr/May). All DNA was extracted from the samples and subjected to shotgun metagenomic sequencing. The resulting data sets were analyzed for the presence of AMR genes. Individual genes detected (shown on *y*-axis) are color coded according to drug class. To allow comparison with the microbiological data set in Figure 2, please note the following antibiotics and their classes: amoxicillin (class: penams), erythromycin (macrolides), trimethoprim (diaminopyrimidines), chlorotetracycline, doxycycline, oxytetracycline (tetracyclines), enrofloxacin, ciprofloxacin (fluoroquinolones), colistin (polymyxins (not detected), neomycin (aminoglycosides), sulfadiazine, and sulfamethoxazole (sulfonamides).

Our metagenomic data set identified a number of resistance genes in aquaculture ponds, with the most frequently detected genes conferring resistance to aminoglycosides, fluoroquinolones, fosfomycins, sulphonamides, and tetracyclines (Figure 3). Some samples contained multiple resistance genes conferring resistance to individual antibiotic classes (in particular sulphonamides), and individual genes were detected that conferred resistance to multiple antibiotic classes (those with multicolored boxes in Figure 3, e.g., SMB-1). Due to the high levels of diversity within the ponds (further discussed below in State 1: Presence of Infectious Disease), we were unable to definitively identify the bacterial hosts of the AMR genes present.

In our social data set, farmers listed a range of antibiotics which they had used previously but no longer use, with the main reason given for stopping use (93% of responses) being ineffectiveness. These included amoxicillin, oxytetracycline, ciprofloxacin, and sulfamethoxazole + trimethoprim (sold in combination; see Table S4 for interview data).

These three data sets were collected through different methods and at different time periods and illustrate the challenges of measuring the burden of AMR in the absence of coordinated surveillance and monitoring systems. Nevertheless, together they provide evidence that AMR is present within Mymensingh rural aquaculture environments.

### Identifying the States, Pressures, and Drivers

Each of the three “States” that create the optimal conditions for AMR emergence and/or transmission in any given environment (see Table S5 for details) was then considered in turn. We used the three data sets, existing literature, and our team’s extensive experience of working in this field to explore the underlying “Pressures” and “Drivers” of each “State” in turn (Table 1). These are discussed below and highlight the substantial fluidity between what constitutes a driver and a pressure in this context, as well as demonstrating the inherent interconnectivity with wider societal issues.

### State 1: Presence of Infectious Disease

Intensification is being actively promoted by the Bangladesh Government’s Department of Fisheries as part of their development strategies^42−44^ but is currently not being supported by corresponding strategies to mitigate for disease and environmental impact, for example, through moving from traditional, extensive practices toward greater biosecurity.^45^ In our social data set, 93% of farmers reported disease in every crop cycle (typically March to December), with an average estimated annual production/profit loss due to disease of 21% (range 5%–35%). Best management practices (BMPs) that help to reduce disease, in particular, fish health management and biosecurity, are generally poor across Bangladesh due to very limited access to training, education, diagnostics and advice, power, affordable credit for investment, governance, and many other factors.^20,46,47^ Only 43% of farmers interviewed had access to a fish disease diagnostic facility. The main symptoms of disease reported were external skin lesions (including hemorrhaging, bubbling of the skin and ulcers), and fin, gill, or tail rot, all indicators of infectious disease and/or stress (Table S4).

Water quality is an issue throughout the season, due to upstream microbial and chemical pollution from industrial, urban, agricultural, and aquaculture sources, and this is worsening with the impacts of climate change.^48^ In July, the monsoon season begins, and by August, high temperatures and heavy surface runoff bring further sediment and pollutants (microbial and chemical/drug pollutants) into aquaculture ponds. In April, temperatures rise quickly as the season transitions from winter to summer, and this is also when farmers introduce fingerlings (juvenile fish) into ponds, so disease outbreaks are more likely as higher temperatures and increasing biomass favor proliferation of many pathogen types.

**Figure 4:**
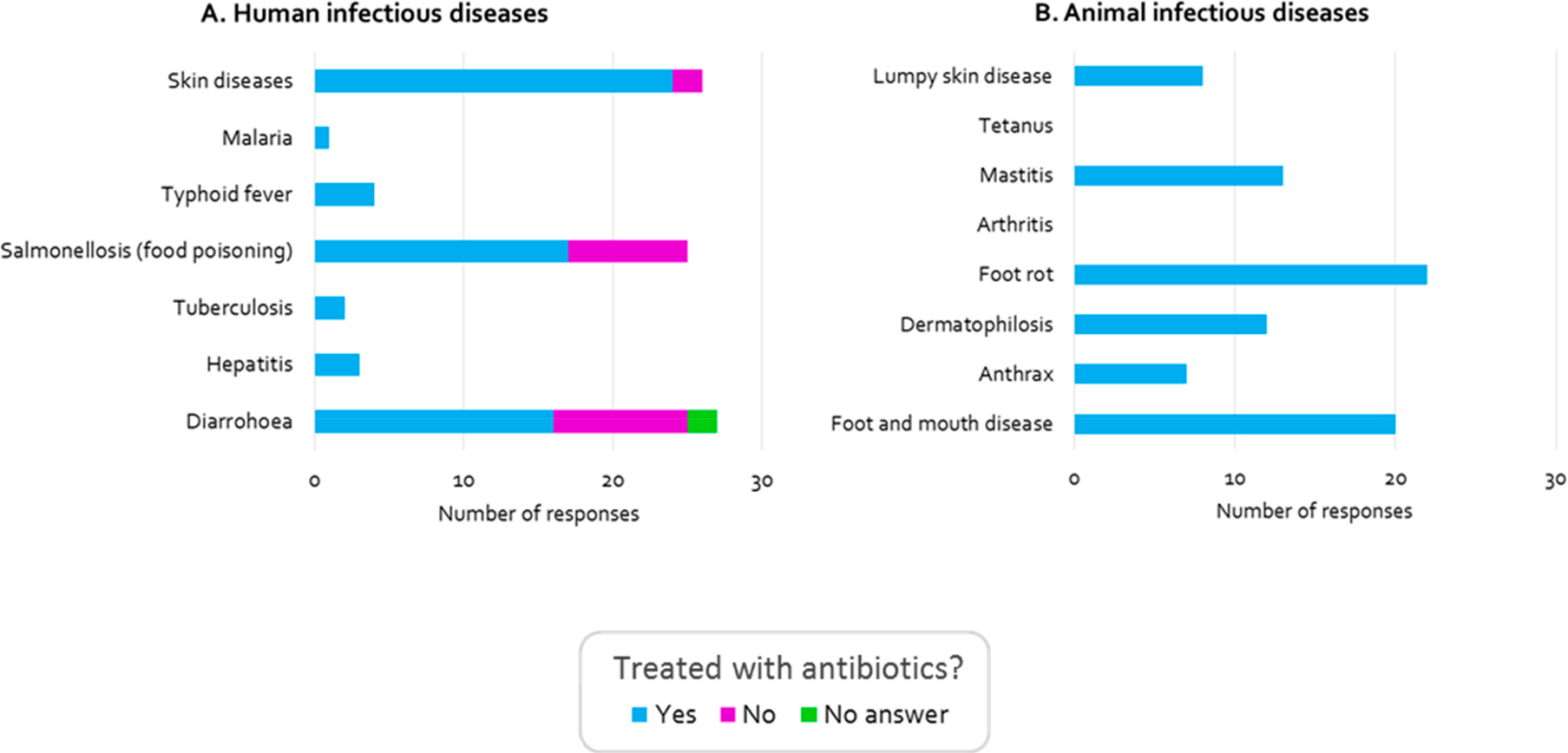
Presence of humans and animal pathogens. In our social data set, farmers were asked whether people (A) and animals (B) on their farm suffer from infectious diseases, as an indication of pathogen presence, and whether these diseases are treated with antibiotics.

Within the rural aquaculture environment, infectious disease is also relatively common among human and other animal inhabitants, largely as a result of limited access to healthcare and sanitation/wastewater treatment (WWT) infrastructure. Ninety-seven percent of farmers reported that people on their farm (family or workers) suffer from infectious diseases, with the most prevalent ones being gastrointestinal and skin diseases (Figure 4). This is in accordance with medical literature, which evidences a high incidence of bacterial and parasitic skin diseases in the Mymensingh region.^49^ Similarly, infectious diseases in other animals within the farm environment were reported by 70% of farmers (Table S4 and Figure 4). Thus, with high levels of infectious disease in humans and animals within the wider farm environment, human and animal pathogens can enter ponds through the use of animal waste as pond fertilizers and through human/animal exposure to ponds (see Opportunities for Human, Animal, or Plant Exposure to Infectious Agents). Fifty-three percent of farmers in our survey used cow dung and poultry droppings to fertilize ponds, and 13 percent of farmers also reported that human waste gets into ponds during the rainy season, as pit latrines flood (Table S4). These are considered poor biosecurity practices, but are common features of traditional farming lifestyles.

In line with the social data set, our metagenomic data set also identified a number of pathogens (including fish pathogens) across all samples collected, but only six at sufficient genomic coverage to reach our confidence cutoff limit for confirming presence (Table S9). *P. aeruginosa* was the only fish pathogen identified. It is also a serious human pathogen, as is *M. tuberculosis*, and these bacteria are established as being widely multidrug resistant and common causes of human disease in Bangladesh.^50,51^ Metagenomic analysis of environmental samples is only likely to detect pathogens present in relatively high numbers, due to the high levels of taxonomic diversity within these environments, and five of these pathogens are environmental bacteria. The exception is *M. tuberculosis*, which is usually considered an obligate parasite (i.e., requires a human host to survive) but is known to survive in anthropogenic environments, including human wastewater.^52^ The detection of *M. tuberculosis* in our metagenomic data set could reflect regular human exposure to these aquaculture ponds, or it could be present due to upstream anthropogenic pollution of the incoming water. Alternatively, this may be an incorrect annotation, resulting from the heavy focus of the pathogen database on human disease and illustrating the limitations of using metagenomics for environmental AMR characterization: mycobacterial diseases of fish are common in aquaculture, and so it is possible that the pathogen identified is an uncharacterized Mycobacterium that is very similar to *Mycobacterium tuberculosis*.

### State 2: Presence of Antimicrobial Compounds

Antibiotic usage is currently poorly governed in the Bangladesh aquaculture sector. As in many LMICs, antibiotics are readily available over the counter across Bangladesh, without a prescription. Aquaculture is currently not addressed in the Bangladesh AMR NAP (Table S1), although we understand that a more comprehensive NAP that includes aquaculture is soon to be released.^53^ The Bangladesh Government is also (as of July 2022) preparing a new *Guideline for Antimicrobial Consumption Surveillance in Bangladesh* that outlines a monitoring framework incorporating both human and animal health sectors.^54^ Regulations on antibiotic usage in Bangladesh aquaculture do exist but are very limited. They are poorly implemented due to insufficient monitoring and surveillance, poor institutional resource capacity (in particular staffing), and poor regulatory control on aggressive and irrational marketing.^55,56^ A 2020 policy from the Government’s Ministry of Fisheries and Livestock banned antibiotic use in food production, with the exception of oxytetracycline and sulfadiazine which are allowed under prescription.^57^ In our social data set, only one farmer could name the antibiotics that are approved for aquaculture use, and none of the farmers kept records of drug use. There was also poor awareness and knowledge on AMR in general and much uncertainty over the recommended withdrawal period (period before harvest when antibiotics should not be used, for food safety reasons). Antibiotic misuse was common among the farmers interviewed in our social data set, with 23% of use reported as prophylactic (prevention of disease), a practice that is widely discouraged and banned in many countries (Table S4). Of the antibiotic treatments reported, 44% were higher than the recommended dosage given on the label, and 66% of farmers did not complete the full course of antibiotics. Some farmers reported use of two different drugs which contained the same active ingredient, and farmers commonly reported using more than one antibiotic within the same time period. In the 12 months prior to interviews, a range of antibiotics were used by farmers, and on average, these accounted for 6% of business costs (Table S4 and Figure S2). Farmers reported that their antibiotic usage was greatest in April and August, which correlates with increasing infectious disease levels due to seasonal climate and water quality variations (see above).

Antibiotics used in aquaculture are most commonly purchased from local farm supply shops. We catalogued 48 different antibiotics being sold by farm shops in the Mymensingh region. Of these, 42 gave information on how to use the drugs on the label, although active ingredient concentrations (and therefore doses) varied between manufacturers, and 6 of the 42 labels included terms promoting prophylactic use. All of the farmers applied antibiotics by mixing with feed (as is recommended), but only 17% followed personal safety precautions when handling antibiotics (Table S4). Commercial aquaculture feed may itself be another route by which antibiotics enter the pond environment, with reports that feed companies are continuing to add antimicrobials to aquaculture feed in order to prolong shelf life, a practice that was banned in Bangladesh through the 2010 Fish Feed and Animal Feed Act.^58,59^ Unused feed and antimicrobial residues can accumulate in pond sludge,^60^ and BMPs stipulate that sludge should be removed at least annually, but all farmers in this study reported removal less often than this. Ninety-three percent of farmers disposed of their sludge onto the pond dykes surrounding the pond, so runoff during rainfall may provide another route for these antimicrobial compounds to re-enter ponds. All farmers sourced their water from underground, which in Bangladesh is widely contaminated with heavy metals and other agrochemical compounds that are known to co-select for AMR.^61,62^ Many aquaculture farmers discharge water into the same local water system, with little or no WWT; thus, pollution from upstream farms (aquaculture and agriculture) can also introduce antimicrobials to the rural aquaculture environment during flooding (Table S4).

Antimicrobials can enter rural aquaculture environments via their use by human and other animal inhabitants in order to treat disease or (in animals) for prophylactic use. Of those reported in our social data set, 76% of human disease cases and 100% of animal disease cases were commonly treated with antibiotics (Figure 4). Antibiotic use among humans in rural areas is higher than that of urban areas in Bangladesh, with drug shops highly prevalent.^63^ We did not collect detailed information on the use of antibiotics in livestock, but prophylactic use, especially in chickens, is a known issue in Bangladesh, with antibiotics commonly added to drinking water despite being banned for use in feed and poultry rearing.^64^ Thus, with a large proportion of antibiotics thought to pass through animals unmetabolized,^65^ residues are likely to be present in the excretory material entering ponds (see above).

### State 3: Opportunities for Human, Animal, or Plant Exposure to Infectious Agents

Traditional farming methods provide many routes for human, animal, and plant exposure to pathogens. Farmers all sold their fish at the local wholesale market, and on average, their household consumed 3% (range 1%–5%) of their production. All farmers reported regular (daily/weekly) human and animal exposure to pond water, for sampling, harvesting, application of drugs and chemicals, observation, and bathing purposes (Table S4). Exposure pathways related to human/animal consumption of vegetation grown within the farm environment were also identified. For example, 77% of farmers used pond water for agricultural farming, and 97% of farmers discharged pond water to rice fields after harvest. Thirty percent of farmers used pond water for rearing animals (drinking, bathing/washing) on a daily basis. Dead animals, which are likely to contain pathogens (and antibiotic residues), were commonly fed to wild animals (reported by 53% of farmers), who may also serve as hosts for pathogen propagation. Some farmers reused dead animals by drying for fish meal (20%) or use as a vegetation fertilizer (13%). Dead animals were also disposed of by throwing “to and fro” (27%), burying (23%), or releasing directly (13%) or indirectly (10%) into local water channels. Due to the open nature of the farms, all farmers reported daily access of wild birds, which have been shown to contribute to AMR dissemination in a number of studies^66,67^ (Table S4).

### Identifying the Responses

**Figure 5:**
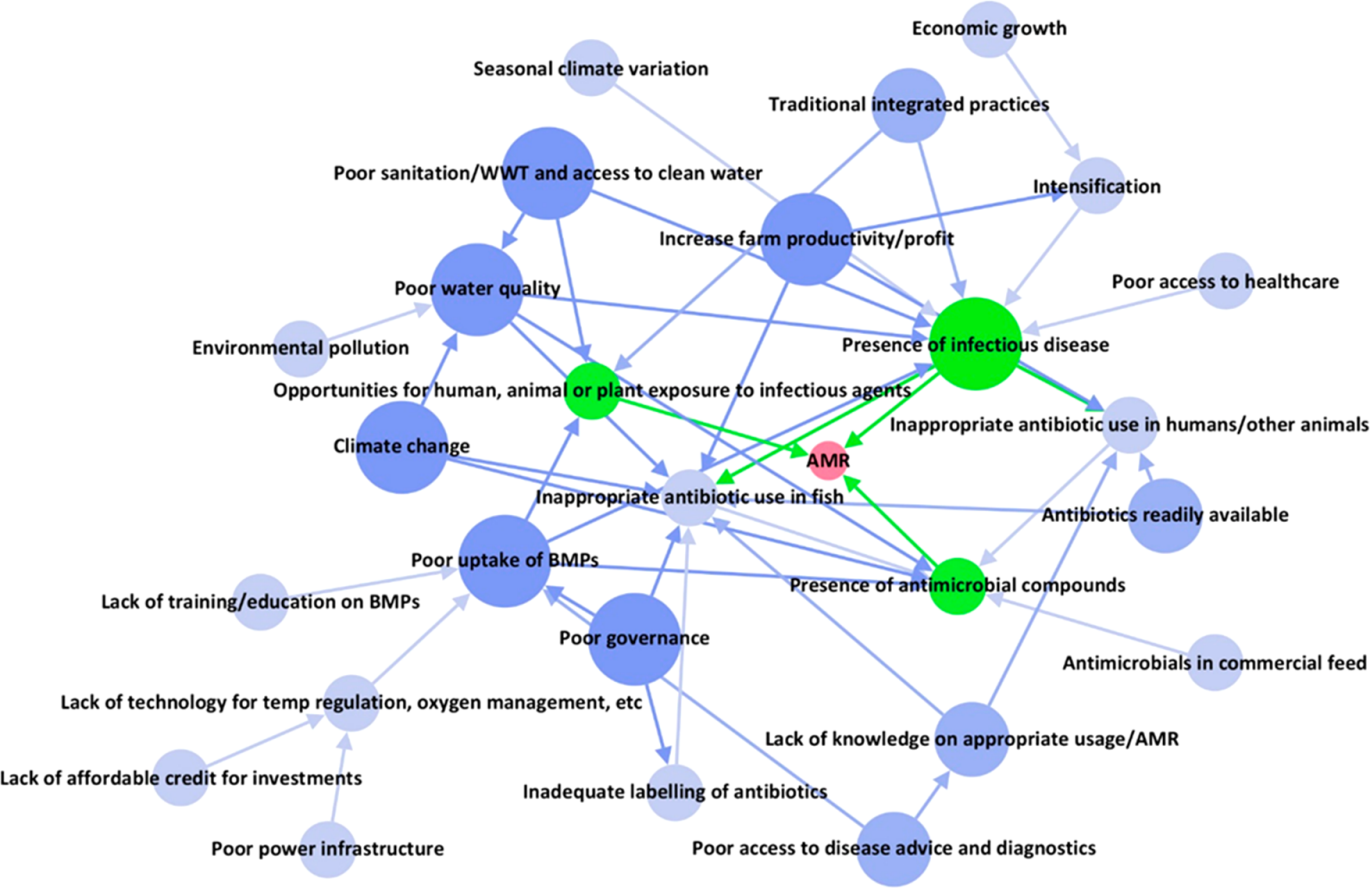
DPSIR network mapping. The DPSIR results (Table 1) were used to generate a network map using Gephi software.^41^ Nodes are scaled according to number of connections in a directional flow from drivers to impact (Figure 1); i.e., larger nodes indicate greater contributions toward AMR. The impact (AMR) is colored in red, states in green, and pressures/drivers in blue, with darker blue indicating greater sizes of nodes. BMP: best management practices. WWT: wastewater treatment.

According to the DPSIR framework (Figure 1), the states, pressures, and drivers described here and listed in Table 1 are potential intervention targets for the “Responses”. We mapped the DPSIR outputs listed in Table 1 using the network mapping software Gephi.^41^ Scaling node size according to the number of forward connections moving through the DPSIR framework, from drivers to impact, shows the factors with the greatest interconnectivity in the issue and which therefore may have the most influence (Figure 5). In terms of the three “States”, this map suggests that the presence of infectious disease is more influential than the presence of antimicrobial compounds or hosts in driving AMR in this environment, as has been demonstrated by other studies looking at factors contributing to AMR.^68^ The DPSIR concept suggests that the strongest interventions are those targeted at the underlying pressures/drivers (Figure 1), and our map indicates that the most influential factors to target (dark blue in Figure 5) would be a range of sustainable development issues affecting not only food production (e.g., poor uptake of BMPs, increase farm productivity/profit) but also the environment (poor water quality, climate change), human health (access to sanitation/WWT and clean water) and, more generally, poor governance. This supports the notion that AMR is intrinsically a sustainable development issue that requires a One Health approach.^6^ It is in agreement with the recommendations from the World Bank and AMR Global Leaders Group, that the most impactful and cost-effective interventions to mitigate AMR in LMICs are likely to be “AMR sensitive” approaches that address AMR indirectly by integrating it into wider sustainable development strategies.^3,7^ Thus, in our application of the DPSIR framework, identifying the “Responses” can be considered as the integration of AMR into these strategies. We identified a number of drivers and pressures which already have policies or initiatives in place in Bangladesh (Table S10).

### Supporting Implementation of AMR policy

This work illustrates the complexity of the issue of AMR in rural LMIC aquaculture environments and supports many other studies on AMR in rural LMIC contexts which have consistently shown that antibiotics are often used as “quick fix” solutions to complex social and economic situations.^69−71^ Effective AMR strategies therefore need to move upstream from surveillance and stewardship of antibiotic usage on a local (farm) level and take a greater focus on a more proactive, preventative approach. This has been shown to be an issue for AMR strategies in general, with global AMR commitments from 2015 to 2021 remaining heavily focused on research, surveillance, and stewardship rather than the more upstream, preventative measures outlined in the 2015 AMR Global Action Plan.^72^ This further speaks to the need for AMR-sensitive approaches and the integration of AMR into wider sustainable development strategies.^5−7^ A recent study evaluating the implementation of the Bangladesh AMR NAP reported a lack of activities targeting AMR in the environmental sector, a shortage of a dedicated and trained workforce, and insufficient funding.^73^ Integrating AMR into wider sustainable development policies may also therefore prove to expedite NAP implementation from an efficiency perspective, through making use of existing funding, resources, and activities targeting the wider sustainable development goals.

Since its development in the 1990s, the DPSIR framework has been applied to a very wide variety of issues and contexts, as both a conceptual and analytical tool.^10,74^ Here, we found that DPSIR was useful as a conceptual framework to help systematically structure our enquiries into the relationships between AMR and the complex underlying social and ecological causal factors in a rural aquaculture environment, but there was considerable fluidity in defining the drivers and pressures. This is also one of the most common discrepancies reported by previous studies using DPSIR^74^ and in our case reflects the intrinsic complexity, interdependency, and synergy between these underlying factors, many of which are sustainable development issues themselves. Other studies have dealt with this real-world complexity by adapting DPSIR to meet their needs, for example, through using a nested DPSIR approach (i.e., multiple DPSIRs that link together)^75^ or reframing DPSIR to incorporate the four spheres of the sustainability model (social, economic, environmental, and political).^76^ Thus, future development of the DPSIR framework for application to AMR in LMIC rural food production environments should examine how to most effectively and appropriately structure these aspects of the framework.

To our knowledge, this is the first study to apply a conceptual framework to a rural LMIC food production AMR case study. It is in line with a growing number of reports emphasizing the importance of tailoring implementation strategies to specific contexts by incorporating local knowledge and expertise and integrating evidence from across different disciplines.^7,77^ In comparison to the more conventional top-down AMR policy implementation methods, we believe that this bottom-up approach, beginning at the community level, offers a more pragmatic route to identifying potential intervention targets, and we believe this is the first study to demonstrate meaningful connectivity between AMR and such a wide range of sustainable development issues. When applying a conceptual framework in this way at a community level, we expect that the value of the outputs will be relative to the quality of the data, knowledge, and expertise available for input and therefore that more valuable outputs (here, more successful long-term intervention strategies) will be gained from engaging with a wide range of local stakeholders in order to gain a perspective of the issue that is as broad and deep as possible. An important challenge here will be how to best ensure objectivity when doing so. Each of these pressures/drivers is in itself a complex issue, so more comprehensive analyses should also better establish how far the boundaries of the framework application should be extended in order to facilitate interconnectivity while containing it enough to maintain practicability as a decision-making tool. Equally, wider discussions on the most appropriate definition of the states used when applying DPSIR to AMR would be useful, as some would argue that commensal microbes, as vectors for AMR transmission, should be included. Inclusion of commensals would blur the boundary between States 1 and 3, by increasing the scope of State 1 (Presence of infectious disease) to include the presence of all human-, animal-, and plant-associated microorganisms (i.e., not just pathogens) and expanding State 3 (Presence of antimicrobial compounds) to include opportunities for exposure not just to humans, animals, and plants but also their commensals. In turn, this would impact on the pressures, drivers, and responses identified.

Overall, this study provides a useful foundation for developing a DPSIR framework that can be applied to AMR in rural LMIC food production environments. Further work is needed to establish the appropriate parameters and categorization of variables within the framework, but once achieved, this approach will provide novel and valuable opportunities to more effectively implement AMR policy and overcome the AMR action gap. Systems modeling techniques could be employed to incorporate the weightings of different factors and support quantification of the broader socioeconomic costs/benefits of different AMR-sensitive interventions.^78^ This would further aid integration into wider sustainable development strategies and support Objective 5 of the AMR Global Action Plan, “to develop the economic case for sustainable investment”, by better understanding and valuing the wider sustainable development goal co-benefits.^5^ We suggest that one or more of the states identified through the DPSIR application could also be used to measure the relative impact of different interventions on AMR, as described in Table S5. This may be a more accessible way of measuring AMR intervention impacts, in the absence of large-scale AMR surveillance and monitoring, so it may be more applicable to an LMIC environmental context.

## Supporting Information Available

Figure S1: Aquaculture production in Bangladesh. Figure S2: Antibiotic usage patterns. Table S1: Top five global aquaculture producers. Table S2: Coordinates of ponds sampled for metagenomic analyses. Table S3: Information from Quality Feed Limited on microbiological analyses. Table S4: Social data set. Table S5: The “States”. Table S6: Files used for Gephi network mapping. Table S7: Proportional antibiotic susceptibility testing data by antibiotic class. Table S8: Antibiotic susceptibility testing data by bacterial genera. Table S9: Presence of pathogens in metagenomic data set. Table S10: Wider Bangladesh policies and initiatives linked to AMR through our DPSIR application. (PDF)
