# Supplementary material for: Antimicrobial Resistance in Aquaculture Environments: Unravelling the Complexity and Connectivity of the Underlying Societal Drivers
Source: Environ Sci Technol. 2022 Sep 14;56(21):14891–903. doi: 10.1021/acs.est.2c00799 (PMC9631993; doi:10.1021/acs.est.2c00799)
Supplement: Supplementary file 1 — es2c00799_si_001.pdf [file es2c00799_si_001.pdf]

SUPPORTING INFORMATION

Antimicrobial resistance in aquaculture environments: unravelling the complexity and connectivity of the underlying societal drivers

Kelly Thornber[1,2*], Abul Bashar[3], Md. Salahuddin Ahmed[4], Ashley Bell[1], Jahcub Trew[1], Mahmudul Hasan[3], Neaz A. Hasan[3], Md. Mehedi Alam[3], Dominique L. Chaput[1], Mohammad Mahfujul Haque[3] and Charles R. Tyler[1,2]

1 – Biosciences, Geoffrey Pope Building, University of Exeter, Stocker Road, Exeter, EX4 4QD, UK
2 – Centre for Sustainable Aquaculture Futures, University of Exeter, Stocker Road, Exeter, EX4 4QD, UK
3 – Department of Aquaculture, Bangladesh Agricultural University, Mymensingh 2202, Bangladesh
4 – Brahmaputra Laboratory, Quality Feed Ltd, Mymensingh 2200, Bangladesh
*Corresponding author

Total number of pages: 27

Total number of Figures: 2

Total number of Tables: 10

Figure S1: Aquaculture production in Bangladesh.

Figure S2: Antibiotic usage patterns.

Table S1: Top five global aquaculture producers.

Table S2: Co-ordinates of ponds sampled for metagenomic analyses.

Table S3: Information from Quality Feed Limited on microbiological analyses.

Table S4: Social dataset. (10 pages)

Table S5: The "States".

Table S6: Files used for Gephi network mapping. (2 pages)

Table S7: Proportional antibiotic susceptibility testing data by antibiotic class.

Table S8: Antibiotic susceptibility testing data by bacterial genera. (4 pages)

Table S9: Presence of pathogens in metagenomic dataset.

Table S10: Wider Bangladesh policies and initiatives linked to AMR through our DPSIR application. (2 pages)

Figure S1: Aquaculture production in Bangladesh. Figure shows map of Bangladesh, with inset illustrating our case study region, the Mymensingh district.

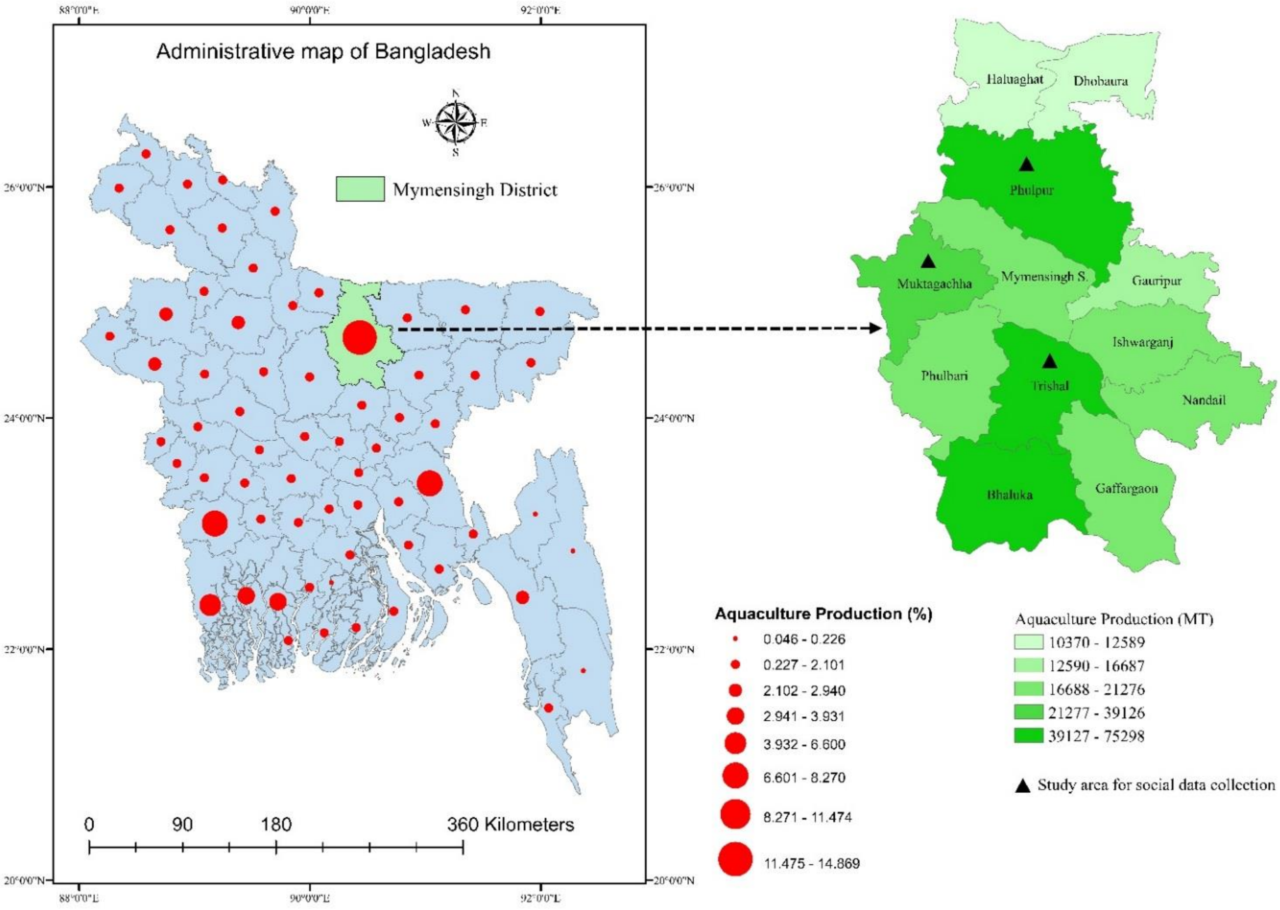

Figure S2: Antibiotic usage patterns. Thirty aquaculture farmers were interviewed in February 2021, and asked which antibiotics they were using. For each antibiotic usage reported, data show **A.** Time of year used. **B.** Length of time the farmer had been using this antibiotic. **C.** Frequency of use.

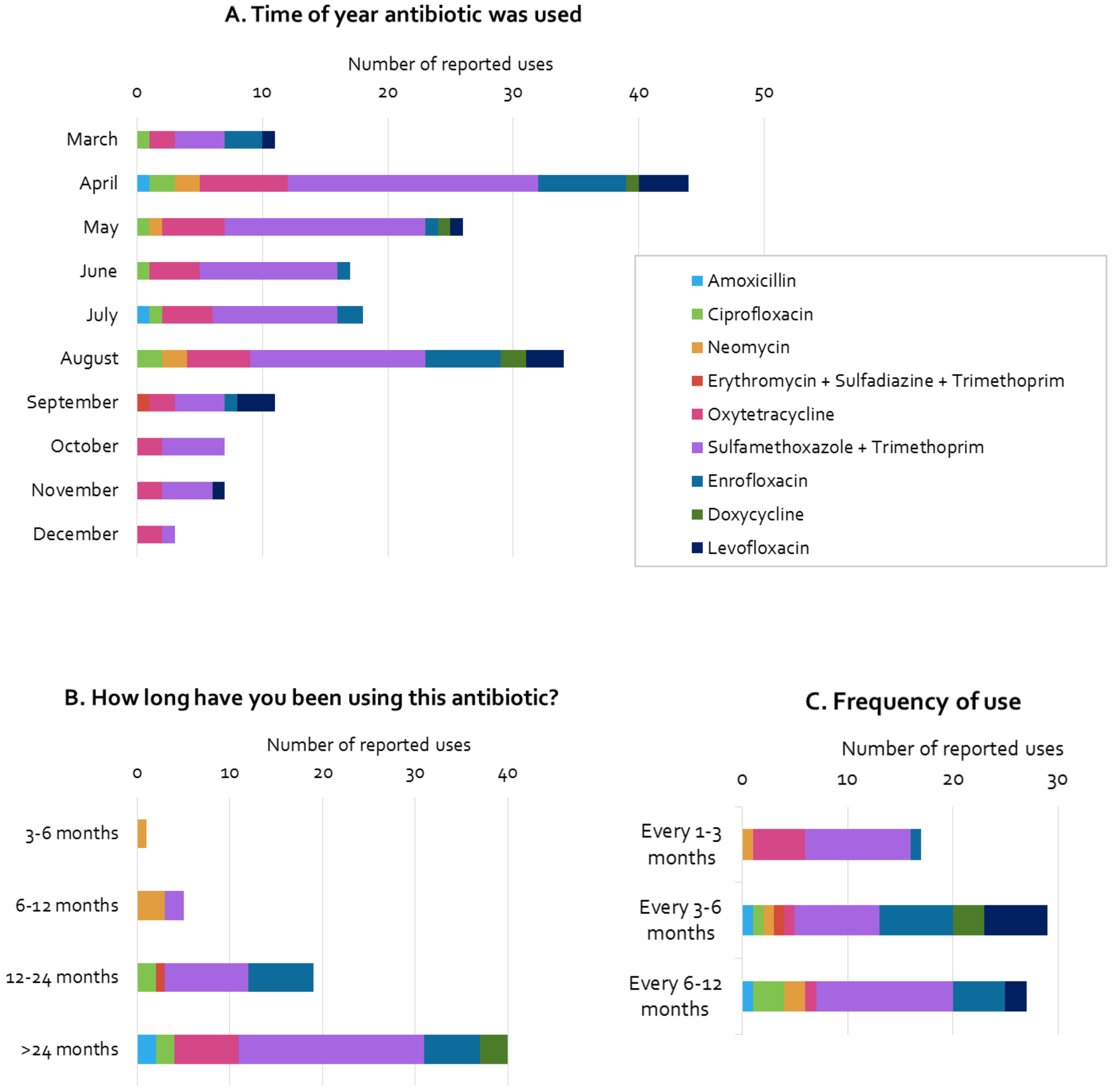

Table S1: Top five global aquaculture producers. Data shown for 2019, taken from the FAO's FishStatJ database. AMR National Action Plans (NAPs) were accessed from the World Health Organisation's Library of AMR NAPs[21].

	**Percentage of global aquaculture production**	**Total aquaculture production (thousand tonnes)**	**Most recent AMR national action plan**	**Does NAP specifically mention aquaculture?**
China	56.97	68,424	2016-2020	No
Indonesia	13.23	15,893	2017-2019	Yes
India	6.49	7,800	2017-2021	Yes
Viet Nam	3.71	4,456	2013-2020	Yes
Bangladesh	2.07	2,489	2017-2022	No

Table S2: Co-ordinates of ponds sampled for metagenomic analyses. Co-ordinates are limited to one decimal point in order to maintain farmer anonymity.

Farmer	Main crop	Village	Upazila	Latitude	Longitude
F1	Pangasius	Kandulia	Muktagacha	24.8	90.3
F2	Pangasius	Kandulia	Muktagacha	24.8	90.3
F3	Pangasius	Malotipur	Muktagacha	24.8	90.3
F4	Tilapia	Bamunpara	Jamalpur_Sadar	24.9	89.9
F6	Tilapia	Gopalpur	Tarakanda	24.8	90.4
F8	Tilapia	Fulbaria	Jamalpur_Sadar	24.9	89.9

Table S3: Information from Quality Feed Limited on microbiological analyses. A. Range of selective media used to incubate initial swabs (information provided by company), and the bacterial genera that they selected for. B. Antibiotic susceptibility testing categorisation. Diameters of zones of inhibition used to define each category. Values used were those of the Clinical and Laboratory Standards Institute Performance Standards[37], with adaptations for neomycin and doxycycline taken from www.microvetdiagnostics.com[64].

A

Media	Genera
BSIGB Agar	*Aeromonas spp.*
MYPA Agar	*Bacillus spp.*
MacConkey Agar	*Citrobacter spp.*
MSA Agar	*Diplococci spp.*
EIM Agar	*Edwardsiella spp.*
ESA Agar	*Enterococcus spp.*
Shieh Agar	*Flavobacterium spp.*
Rogosa Agar	*Lactobacillus spp.*
Cetrimide Agar	*Pseudomonas spp.*
MSA Agar	*Staphylococcus spp.*
Strept Selection Agar	*Streptococcus spp.*
TCBS Agar	*Vibrio spp.*

B

Antibiotic disc	Resistant (mm)	Intermediate susceptibility (mm)	Sensitive (mm)
Amoxicilin	≤13	14-17	≥18
Ciprofloxacin	≤15	16-20	≥21
Colistin	≤8	9-10	≥11
Doxycycline	≤12	13-15	≥16
Erythromycin	≤13	14-22	≥23
Trimethoprim	≤10	11-15	≥16
Chlortetracycline	≤14	15-18	≥19
Enrofloxacin	≤14	15-17	≥18
Neomycin	≤12	13-16	≥17
Levofloxacin	≤13	14-16	≥17

Table S4: Social dataset. Data collected in February 2021 from interviews from 30 farmers representative of the wider aquaculture community in the region.

SECTION A: ABOUT YOU AND YOUR FARM

Question	Results (From 30 responses)
Age	Average = 43.3 years
Gender	100% male
Farm location	6 Jamalpur<br>3 Fulbaria<br>3 Gopalpur<br>5 Trishal<br>6 Phulpur<br>6 Muktagacha<br>1 Gohakandi
Education level	3 No education<br>8 Primary<br>15 Secondary<br>1 Higher secondary<br>3 University
Role on farm	28 Owners<br>2 Managers
Time spent in aquaculture	Average = 12.3 years
Size of farm (Ha)	Average = 1.76
Number of ponds	Average = 5.3
Species stocked	(All polyculture)<br>18 Pangas<br>28 Tilapia<br>20 Shing<br>15 Pabda<br>9 Gulsha<br>28 Carps<br>4 Magur<br>1 Bangla<br>1 Koi

SECTION B: DISEASE MANAGEMENT AND ANTIMICROBIAL USAGE

Question 1: Please indicate how much the following factors threaten the profitability of your business (out of 5)

	Poor water quality	Poor environmental (soil and water) quality	Poor access to bank loan	Low market price	Disease	Poor access to diagnostics	High cost of drugs/chemicals	Poor access to good quality drugs/chemicals	Poor access to advice on drug/chemical usage	Drug/chemical ineffectiveness	Having to comply with withdrawal period	Poor access to training	Flooding and other natural events	Uncontrollable run-off during monsoon season
Average	3.4	2.6	1.2	5.0	4.6	2.9	2.8	2.6	3.0	3.0	1.9	2.3	1.8	1.7

Question 2: Frequency of disease outbreak?

Every crop cycle	27
Irregular interval	1
Not occurred	1

Question 3: Which sign and symptoms (diseases) are most frequent on your farm?

Fin, gill and/or tail rot	32
External skin lesions including haemorrhaging, bubbling of the skin and ulcers	53
Excessive skin mucus production	2
Skin discolouration	4
Exopthalmia (bulging eye sockets)	15
Ascitis (swelling of the abdomen)	13
External parasites (e.g. argulus)	4
Abnormal behaviours – including erratic movements, inability to hold position in the water column, gulping for air, reduced feeding, etc.	19
Rectal protrusion	15

Question 4: What proportion of your annual production/ profit, are impacted by disease?
Average = 21%

Question 5: Do you have access to any disease diagnosis facility?

Yes	13	
No	17	
If yes, which facilities	BAU disease lab	10
	Quality Feed Ltd Aqua Lab	3
Barriers to using them	Time consuming	4
	Distance	8
	No answer	1
How often useful (average)		61%

Table S4

Question 6: Which of the following pharmaceuticals have you used in the past 12 months?

For each antibiotic used, the following questions were asked:	Number of responses
Active ingredient (determined by our Team members, based upon the brand name given)	
Amoxicillin	2
Ciprofloxacin	3
Neomycin	4
Ery + Sulf + Trimeth	1
Oxytetracycline	7
Sulf + Trimeth	30
Enrofloxacin	13
Doxycycline	3
Levofloxacin	7
Frequency of use	
Every 1-3 months	17
Every 3-6 months	29
Every 6-12 months	27
What time of year did you use this?	
March	11
April	44
May	23
June	14
July	15
August	34
September	11
October	7
November	7
December	4
How long have you been using this?	
3-6 months	1
6-12 months	5
12-24 months	23
>24 months	45

Question 6 (continued)

For each antibiotic used, the following questions were asked:	Number of responses
Reasons for use?	
Control	56
Prevention	13
Both	4
Was it effective?	
Yes	42
No	31
Mode of application	
Mixed with feed	73
Dosage used (determined by our Team: dosage given by the farmer was compared to that given on product label)	
High	32
Recommended	41
Low	0

Question 7: What proportion of your business costs are used on the drugs listed above?
Average = 6%

Question 8: Do you purchase and use antibiotics based on prescription?
Yes 15
No 15
If not, what prevents you from obtaining a prescription?
No technical person 8
Prescription not effective* 6
No answer 1
*[*Infers that farmers did not believe the prescriptions would be effective]*

Question 9: Do you complete the full course of the drugs listed above in ponds?
Yes 10
No 20
If not, why?
Costly 5
Recommended dose is not effective 15

Question 10: Do you maintain the recommended withdrawal period?
Yes 11
No 19
If not, why?
Fish become cured 4
Fish need to be sold 12
Costly 5

Question 11: Are there any of the drugs above that you feel have become less effective over time?

Yes	25
No	5

Question 12: Are there any of the drugs above that you have used previously but no longer use?

Amoxicillin	21
Ciprofloxacin	15
Neomycin	4
Erythromycin + Sulfadiazine + Trimethoprim	5
Oxytetracycline	18
Sulfamethoxazole + Trimethoprim	10
Enrofloxacin	5
Doxycycline	3

Reasons for stopping use

Ineffective	30
Costly	9
Not available	18

Question 13: Do you keep records of drug use for your farm?

Yes	0
No	30

SECTION C: HUMAN AND LAND ANIMAL EXPOSURE TO ANTIMICROBIALS AND ANTIMICROBIAL-POLLUTED WATER

Question 1: Where do you sell your fish?

Wholesale market	30

Question 2: What proportion of your fish do you/your family consume?
Average = 3%

Question 3: Do you use pond water in rearing animals (cattle, poultry and others)?

Yes	9
No	21

If yes, please give details on how it is used (e.g. washing, drinking) and how often.

Drinking	9
Bathing/washing*	7
Daily	9

*[*Infers bathing/washing of animals by farmers]*

Question 4: Do you use pond water in agricultural farming (rice, vegetables, fruit)?

Yes	23
No	7

If yes, please give details on how it is used (e.g. washing, drinking) and how often.

Irrigating rice field	15
Watering vegetables	11
Daily	1
Weekly	9
Monthly	15

Question 5: Is there any access for wild birds to pond water? If so how often.

Yes	30
Daily	30

Question 6: Do you use cow-dung and poultry-dropping in your ponds?

Yes	16
No	14
If yes, how often?	
Weekly	3
Fortnightly	1
Monthly	11
Rarely	1

Question 7: Does human and household waste find their way to your pond?

Yes	4
No	26
If yes, what types of waste and how often does this enter the pond?	
Household waste, during rainy season	4

Question 8: How do you use/mix antimicrobials with feeds or water?

Hand mix (feed/water)	30
Direct pouring (water)	21

Question 9: Do you follow any precautionary measure during application of antimicrobials?

Yes	5
No	25
If Yes, which of the following?	
Mask	5

Question 10: Please explain how the different people on your farm (farmers/workers/others) have contact with pond water:

Person on farm	*No. responses*	*Purpose*	*No. responses*	*Frequency*	*No. responses*
Farmer	30	Sampling	30	Daily	1
		Harvesting	30	Weekly	16
		Drug/chemical application	30	Monthly	13
		Observation	17		
		Bathing	3		
Farmer's wife	2	Household work	2		
Children	16	Sampling	12	Daily	
		Harvesting	12	Weekly	10
		Drug/chemical application	1	Monthly	6
		Bathing	8		
Farm worker	30	Sampling	30	Daily	15
		Harvesting	30	Weekly	15
		Drug/chemical application	30	Monthly	
		Observation	25		
		Bathing	9		
		Other farming tasks	2		

Question 11: Do any persons on your farm (family or workers) suffer from infectious diseases?

Yes	29
No	1

If yes, please name specific ones	*Total*	*Is this usually treated with antibiotics?*		
		Yes	*No*	*No answer*
Diarrohoea	27	16	9	2
Hepatitis	3	3	0	0
Tuberculosis	2	2	0	0
Salmonellosis (food poisoning)	25	17	8	0
Typhoid fever	4	4	0	0
Malaria	1	1	0	0
Skin diseases	26	24	2	0

Question 12: Do any animals on your farm suffer from infectious diseases?

Yes	21
No	8
No answer	1

If yes, please name specific ones	*Total*	*Is this usually treated with antibiotics?*		
		Yes	*No*	*No answer*
Foot and mouth disease	20	20	0	0
Anthrax	7	7	0	0
Dermatophilosis	12	12	0	0
Foot rot	22	22	0	0
Arthritis	0	0	0	0
Mastitis	13	13	0	0
Tetanus	0	0	0	0
Lumpy skin disease	8	8	0	0

SECTION D: ANTIMICROBIAL/PHARMACEUTICAL POLLUTION AND THE ENVIRONMENT

Question 1: What are the main sources of water for your farm?

Underground water	30
Rainfall	19

Question 2: Are there any possible water contamination sources?

Yes	1
No	29

Question 3: How frequently do you dry ponds?

After each cycle	0
Once/year	24
Less frequent	6

Question 4: How frequently do you remove sludge?

After each cycle	0
Once/year	0
Less frequent	30

Question 5: Where do you use or dispose of the removed sludge?

Agricultural field	1
Rice field	4
Pond dyke	28
Low land	9
Canal/beel	1

Question 6: After harvesting fish, where do you discharge the pond water?

Rice field	29
Vegetables land	1
Canal	4
Low land	1

Question 7: How do you dispose of dead animals?

Buried	7
Feed to wild animals	16
Pond dyke	3
Canal	4
Dry for fish meal	6
Throw to and fro	8
Use as fertiliser	4

Question 8: Do you take any measure to treat diseased pond water before discharging to the environment?

Never	30

Question 9: Do you take any measure to treat non-diseased pond water before discharging to the environment?

Never	30

Question 10: Where do you discharge bottles or packs of antimicrobials after using?

Burnt	2
Throw to and fro	14
Rice fields	10
Ponds	8
Pond dykes	1
Gather to sell	4
Canal	4

Question 11: Do you use any measure before discarding these bottles or packs?

Yes	2 (burning)
No	28

Question 12: How often does your farm flood?

Once/year	8
Rarely	6
Never	16

**Question 13:** Does flooding impact on your need to use pharmaceuticals?	
Yes	13
No	17
If yes, please give details	
Increase disease outbreak	5
Water quality deterioration	7
Reduced water transparency	3
Waste water enters which requires more disinfectants	6

**Question 14:** Do you think aquafarming negatively impacts on the environment?	
Yes	1
No	29
If yes, how?	
Water discharge pollute environment	1

**Question 15:** Have you noticed any changes to the environment quality (water, soil, air) during the time you have been farming?	
Yes	11
No	19
If yes, what do you think are the main causes of this?	
Decomposition of excess unused feed	7
Intensification reduces the soil quality	1
Rice field fertility increased	1
Use of chemicals/drugs for long time	2
Crop land besides pond give lower production	1

**Question 16:** Do you follow any strategy to lessen the environmental impacts of your farm?	
Yes	0
No	30

**Question 17:** How important is preserving the local environment to you?	
Not important	0
Slightly important	5
Important	23
Very important	2

SECTION E: KNOWLEDGE AND ATTITUDES TOWARDS ANTIMICROBIALS AND ANTIMICROBIAL RESISTANCE

**Question 1:** Have you heard of any of the following terms:	
Antibiotic resistance	5
Superbugs	0
Antimicrobial resistance	4
AMR	3
Drug resistance	8
Antibiotic-resistant bacteria	2
**Only 10 farmers in total had heard of any of these terms; those that had often listed multiple*	
If yes to any of the above, do you think this is a problem that will affect you?	
Yes	5
No	5

Question 1 (continued)

What do you think is causing this issue?	
No idea	1
Irresponsible use of drugs	4
Bacteria become stronger	3
Antibiotics become ineffective with diseases	1
No answer	1

Question 2: What is the recommended withdrawal period of antimicrobials before harvest?

No idea	7
3 days	4
7 days	6
14 days	4
15 days	3
20 days	2
30 days	4

Question 3: Which antibiotics are approved for aquaculture use in the national drug policy and/or guideline for aquatic animal disease?

No idea	29
Oxytetracycline and Sulfadiazine	1

Question 4: True/False

	TRUE	FALSE	NO IDEA
Antibiotic resistance occurs when humans or animals become resistant to antibiotics and they no longer work as well	9	1	20
Many infectious diseases are becoming increasingly resistant to treatment by antibiotics	20	0	10
If bacteria are resistant to antibiotics, it can be very difficult or impossible to treat the infections they cause	12	4	14
Antibiotic resistance is an issue that could affect me or my family	5	18	7
Antibiotic resistance is an issue in other countries but not here	11	9	10
Antibiotic resistance is only a problem for people who use antibiotics regularly	16	5	9
Bacteria which are resistant to antibiotics can be spread from person to person, animal to animal or pond to pond	2	2	26
Antibiotic-resistant infections could make medical procedures like surgery, organ transplants and cancer treatment much more dangerous	4	2	24

Table S5: The "States". Table shows how each state could provide optimal conditions for AMR emergence and dissemination, and how they could be measured in LMIC environments in the absence of technical assays. HGT: horizontal gene transfer.

**State**	**How does this support the emergence and spread of AMR?**	**What could be measured?**
Presence of infectious disease	If pathogens (microbes conferring disease) are present in the environment, they can receive resistance genes through HGT and become resistant themselves.<br>*We have not included commensals (non-pathogenic microbes that live on or in organisms) in the definition of this state, as i) their contribution towards AMR is indirect, by receiving and then transferring resistant genes to pathogenic microbes; ii) the presence of commensals and their carriage of resistant genes is more difficult and less frequently assessed through traditional microbiological and social data collection, which are more accessible than genomic methods in LMICs; and iii) the rate of transfer of AMR genes from different commensals to different pathogens remains uncertain.	Accurate: Levels of human, animal and plant pathogens within the aquaculture environment, e.g. through microbiological/genomic techniques.<br><br>Alternative: Levels of infectious disease cases could be measured. Pathogens may be present in the absence of disease in healthy individuals, however the presence of disease is typically associated with higher levels of pathogens within an environment and so could be used as a measure in the absence of access to these techniques.
Presence of antimicrobial compounds	The presence of antimicrobial compounds drives microbes to express resistance genes, and to share them with other microbes through HGT.	Accurate: Levels of antimicrobials within the aquaculture environment, e.g. through spectrometry, spectroscopy, microfluidics, immunoassays.<br><br>Alternative: Levels of application/administration of antimicrobials within the aquaculture environment. The availability of antimicrobial compounds is affected by their individual physiocochemical properties (e.g. solubility in water, adsorption to soil, half-life, etc) and patterns of metabolism, but generally the more frequently they are used the greater the environmental concentrations are likely to be.
Opportunities for human, animal or plant exposure to infectious agents	If a pathogen comes into contact with a human, animal or plant host, it can infect and multiply rapidly, especially if the host has a weak immunity (e.g. due to stress, poor health). This in turn increases the risk of these pathogens (and any associated resistance) being spread to other hosts.	Frequency and duration of human, animal or plant exposure to the aquaculture environment

Table S6: Files used for Gephi network mapping. The attributes.csv (A) and edgelist.csv (B) files used to input into Gephi software are shown as tables.

A.

Id	Label
1	Presence of infectious disease
2	Presence of antimicrobial compounds
3	Opportunities for human, animal or plant exposure to infectious agents
4	Intensification
5	Traditional integrated practices
6	Poor uptake of BMPs
7	Poor water quality
8	Seasonal climate variation
9	Poor access to healthcare
10	Poor sanitation/WWT and access to clean water
11	Inappropriate antibiotic use in fish
12	Antimicrobials in commercial feed
13	Inappropriate antibiotic use in humans/other animals
14	Economic growth
15	Increase farm productivity/profit
16	Lack of training/education on BMPs
17	Poor access to disease advice and diagnostics
18	Poor governance
19	Lack of technology for temp regulation, oxygen management, etc
20	Environmental pollution
21	Climate change
22	Lack of knowledge on appropriate usage/AMR
23	Presence of infectious disease
24	Antibiotics readily available
25	Inadequate labelling of antibiotics
26	Lack of affordable credit for investments
27	Poor power infrastructure
28	AMR

B.

Source	Target	Type	Weight
4	1	Directed	1
5	1	Directed	1
6	1	Directed	1
7	1	Directed	1
8	1	Directed	1
9	1	Directed	1
10	1	Directed	1
11	2	Directed	1
12	2	Directed	1
13	2	Directed	1
6	2	Directed	1
5	3	Directed	1
6	3	Directed	1
10	3	Directed	1
16	6	Directed	1
17	6	Directed	1
18	6	Directed	1
19	6	Directed	1
20	7	Directed	1
10	7	Directed	1
21	7	Directed	1
18	11	Directed	1
22	11	Directed	1
1	11	Directed	1
15	11	Directed	1
21	11	Directed	1
7	11	Directed	1
24	11	Directed	1
25	11	Directed	1
1	13	Directed	1
15	13	Directed	1
24	13	Directed	1
22	13	Directed	1
26	19	Directed	1
27	19	Directed	1
17	22	Directed	1
18	25	Directed	1
1	28	Directed	1
2	28	Directed	1
3	28	Directed	1
21	2	Directed	1
7	2	Directed	1
14	4	Directed	1
15	4	Directed	1

Table S7: Proportional antibiotic susceptibility testing data by antibiotic class. Table shows the data presented in Figure 2, as the proportion of isolates that showed sensitivity (S), intermediate resistance (I) or resistance (R) to the antibiotics listed. Blank cells indicate not tested.

		Oct-19	Nov-19	Dec-19	Jan-20	Feb-20	Mar-20	Apr-20	May-20	Jun-20	Jul-20	Aug-20	Sep-20
**No. isolates tested per month**		15	6	10	13	25	27	26	16	20	26	28	23
**Amoxicillin**	**S**	6.7	0.0	0.0	7.7	0.0	0.0	0.0	0.0	0.0	0.0	0.0	0.0
	**I**	0.0	0.0	0.0	0.0	0.0	0.0	3.8	0.0	0.0	3.8	0.0	0.0
	**R**	93.3	100.0	100.0	92.3	100.0	100.0	96.2	100.0	100.0	96.2	100.0	100.0
**Ciprofloxacin**	**S**	100.0	100.0	60.0	84.6	60.0	48.0	36.0	71.4	50.0	50.0	39.3	26.1
	**I**	0.0	0.0	0.0	15.4	40.0	52.0	64.0	28.6	50.0	50.0	60.7	73.9
	**R**	0.0	0.0	40.0	0.0	0.0	0.0	0.0	0.0	0.0	0.0	0.0	0.0
**Chlortetracycline**	**S**	0.0	66.7	33.3	25.0	4.2	3.8	0.0	0.0	0.0			0.0
	**I**	0.0	33.3	0.0	41.7	58.3	57.7	73.9	70.0	33.3			30.8
	**R**	0.0	0.0	66.7	33.3	37.5	38.5	26.1	30.0	66.7			69.2
**Colistin**	**S**	100.0	100.0	90.0	61.5	4.0	3.8	4.8	6.7	0.0	0.0	0.0	0.0
	**I**	0.0	0.0	10.0	38.5	88.0	84.6	85.7	93.3	81.3	100.0	95.0	100.0
	**R**	0.0	0.0	0.0	0.0	8.0	11.5	9.5	0.0	18.8	0.0	5.0	0.0
**Doxycycline**	**S**	78.6	100.0	60.0	33.3	8.3	11.5	3.8	18.8	5.6	7.7	3.8	13.6
	**I**	14.3	0.0	0.0	58.3	62.5	73.1	69.2	81.3	88.9	84.6	92.3	72.7
	**R**	7.1	0.0	40.0	8.3	29.2	15.4	26.9	0.0	5.6	7.7	3.8	13.6
**Enrofloxacin**	**S**		100.0	33.3	61.5	12.5	42.3	16.7	6.3	0.0	11.1	0.0	0.0
	**I**		0.0	0.0	30.8	79.2	46.2	66.7	93.8	90.0	77.8	100.0	81.8
	**R**		0.0	66.7	7.7	8.3	11.5	16.7	0.0	10.0	11.1	0.0	18.2
**Erythromycin**	**S**	6.7	0.0	20.0	7.7	0.0	0.0	0.0	0.0	0.0	0.0	0.0	0.0
	**I**	53.3	16.7	20.0	30.8	36.0	15.4	11.5	18.8	5.0	23.1	3.6	0.0
	**R**	40.0	83.3	60.0	61.5	64.0	84.6	88.5	81.3	95.0	76.9	96.4	100.0
**Levofloxacin**	**S**								100.0	60.0	34.8	53.6	56.5
	**I**								0.0	40.0	65.2	46.4	43.5
	**R**								0.0	0.0	0.0	0.0	0.0
**Neomycin**	**S**								100.0	33.3	0.0	0.0	0.0
	**I**								0.0	66.7	100.0	100.0	100.0
	**R**								0.0	0.0	0.0	0.0	0.0
**Trimethoprim**	**S**	86.7	83.3	60.0	61.5	13.0	21.7	40.0	6.3	10.0	4.3	0.0	0.0
	**I**	0.0	0.0	0.0	30.8	87.0	73.9	60.0	75.0	75.0	52.2	28.6	47.8
	**R**	13.3	16.7	40.0	7.7	0.0	4.3	0.0	18.8	15.0	43.5	71.4	52.2

Table S8: Antibiotic susceptibility testing data by bacterial genera. Microbiological dataset shown in Figure 2, presented in terms of resistance according to bacterial genera. Data shows the proportion of isolates that showed sensitivity (S), intermediate resistance (I) or resistance (R) to the antibiotics listed.

		Aeromonas			Edwardsiella			Flavobacterium			Pseudomonas			Staphylococcus			Streptococcus			Vibrio			Citrobacter			Enterococcus			Diplococci			Bacillus			Lactobacillus		
		S	I	R	S	I	R	S	I	R	S	I	R	S	I	R	S	I	R	S	I	R	S	I	R	S	I	R	S	I	R	S	I	R	S	I	R
Oct-19	Amoxicillin			4			3			1	1		2									3															1
	Ciprofloxacin	4			3			1			3									3															1		
	Colistin	4			3			1			3									3															1		
	Doxycycline	3			2			1			1		2							3															1		
	Erythromycin			1			1			1			2							1																	1
	Trimethoprim	4			3			1			1		2							3															1		
	Chlortetracycline																																				
	Enrofloxacin																																				
	Levofloxacin																																				
	Neomycin																																				
Nov-19	Amoxicillin			3						1			1									1															
	Ciprofloxacin	3						1			1									1																	
	Colistin	3						1			1									1																	
	Doxycycline	3						1			1									1																	
	Erythromycin			3						1			1																								
	Trimethoprim	2		1				1			1									1																	
	Chlortetracycline	1									1																										
	Enrofloxacin										1																										
	Levofloxacin																																				
	Neomycin																																				
Dec-19	Amoxicillin			2						1			3									3			1												
	Ciprofloxacin			2				1			1		2							3			1														
	Colistin	2						1			3									2			1														
	Doxycycline			2				1			1		2							3			1														
	Erythromycin			2						1			2							2					1												
	Trimethoprim			2				1			1		2							3			1														
	Chlortetracycline			2									2							1			1														
	Enrofloxacin			2									2							1			1														
	Levofloxacin																																				
	Neomycin																																				

		Aeromonas			Edwardsiella			Flavobacterium			Pseudomonas			Staphylococcus			Streptococcus			Vibrio			Citrobacter			Enterococcus			Diplococci			Bacillus			Lactobacillus		
Continued		S	I	R	S	I	R	S	I	R	S	I	R	S	I	R	S	I	R	S	I	R	S	I	R	S	I	R	S	I	R	S	I	R	S	I	R
Jan-20	Amoxicillin			6						1			2	1					1						1									1			
	Ciprofloxacin	5						1			2			1			1						1														
	Colistin	3						1						1			1						1									1					
	Doxycycline	1									1			1			1								1												
	Erythromycin	1		4									1						1						1									1			
	Trimethoprim	3						1			1			1			1						1											1			
	Chlortetracycline	1		1						1	1			1											1									1			
	Enrofloxacin	1		1				1			2			1			1						1									1					
	Levofloxacin																																				
	Neomycin																																				
Feb-20	Amoxicillin						1						6			1			4			2						1			1						
	Ciprofloxacin										4						3			2									1								
	Colistin																		1																		
	Doxycycline												2						1												1						
	Erythromycin						1						5			1			2			1						1									
	Trimethoprim										1															1											
	Chlortetracycline												2			1	1					1									1						
	Enrofloxacin				1						1			1																							
	Levofloxacin																																				
	Neomycin																																				
Mar-20	Amoxicillin			12			1			1			2						7			2			1			1									
	Ciprofloxacin	6			1			1									3			1																	
	Colistin			2															1				1														
	Doxycycline			2									2				3																				
	Erythromycin			10			1			1			2						4			2			1			1									
	Trimethoprim	3															1			1					1												
	Chlortetracycline			5									2				1					2						1									
	Enrofloxacin	6		1							2						2					1			1	1											
	Levofloxacin																																				
	Neomycin																																				

		Aeromonas			Edwardsiella			Flavobacterium			Pseudomonas			Staphylococcus			Streptococcus			Vibrio			Citrobacter			Enterococcus			Diplococci			Bacillus			Lactobacillus		
Continued		S	I	R	S	I	R	S	I	R	S	I	R	S	I	R	S	I	R	S	I	R	S	I	R	S	I	R	S	I	R	S	I	R	S	I	R
Apr-20	Amoxicillin			13			3			3			4			1						1															
	Ciprofloxacin	5									2			1						1																	
	Colistin			2							1																										
	Doxycycline	1		4			1						2																								
	Erythromycin			12			3			1			4			1			1			1															
	Trimethoprim	4			3			3																													
	Chlortetracycline			3			1						1									1															
	Enrofloxacin	3		4													1																				
	Levofloxacin																																				
	Neomycin																																				
May-20	Amoxicillin			3			1			4			4						1			2			1												
	Ciprofloxacin	2						4			2									2																	
	Colistin																						1														
	Doxycycline	1									1						1																				
	Erythromycin			3			1			3			4									1			1												
	Trimethoprim			1	1					1			1																								
	Chlortetracycline			1									1									1															
	Enrofloxacin										1																										
	Levofloxacin				1			2																													
	Neomycin				1			2									1																				
Jun-20	Amoxicillin			10			4			3			1									1			1												
	Ciprofloxacin	6			3															1																	
	Colistin			1			2																														
	Doxycycline	1		1																																	
	Erythromycin			10			4			2			1									1			1												
	Trimethoprim			2	1		1																1														
	Chlortetracycline			1			1																														
	Enrofloxacin			2																																	
	Levofloxacin	5						3			1																										
	Neomycin	1			1															1																	

		Aeromonas			Edwardsiella			Flavobacterium			Pseudomonas			Staphylococcus			Streptococcus			Vibrio			Citrobacter			Enterococcus			Diplococci			Bacillus			Lactobacillus		
Continued		S	I	R	S	I	R	S	I	R	S	I	R	S	I	R	S	I	R	S	I	R	S	I	R	S	I	R	S	I	R	S	I	R	S	I	R
Jul-20	Amoxicillin			8			4			6			5						2																		
	Ciprofloxacin	5			2			2			3						1																				
	Colistin																																				
	Doxycycline			2				1			1																										
	Erythromycin			8			3			3			5						1																		
	Trimethoprim			4			1	1		2			3																								
	Chlortetracycline																																				
	Enrofloxacin	1		1	1								1																								
	Levofloxacin	2			1			3			1						1																				
	Neomycin																																				
Aug-20	Amoxicillin			8			7			5			3			1						2			1			1									
	Ciprofloxacin	2			4			2			1			1						1																	
	Colistin						1																														
	Doxycycline			1																			1														
	Erythromycin			7			7			5			3			1						2			1			1									
	Trimethoprim			6			5			5			1									2						1									
	Chlortetracycline																																				
	Enrofloxacin																																				
	Levofloxacin	5			3			3			2									1						1											
	Neomycin																																				
Sep-20	Amoxicillin			11			1			4			4									1						1									
	Ciprofloxacin	3									2																										
	Colistin																																				
	Doxycycline	1		3				1																		1											
	Erythromycin			11			1			4			4									1						1									
	Trimethoprim			3			1			4			4																								
	Chlortetracycline			5						1			2									1															
	Enrofloxacin			3			1																														
	Levofloxacin	7			1			3			1									1																	
	Neomycin																																				

Table S9: Presence of pathogens in metagenomic dataset. Our metagenomic DNA sequences were classified using Kraken 2 and filtered using the American Biological Safety Association pathogen database (ABSA). Positive identification was taken as 50% coverage. Six pathogens were detected in sufficient quantities to achieve 50% coverage across our sample set. A/B/C/D refer to their presence at the different timepoints sampled, with A = monsoon (Jul/Aug), B = post monsoon (Oct/Nov), C = winter (Jan/Feb), D = pre-monsoon (Apr/May).

Pathogen name	Description	Pond 1	Pond 2	Pond 3	Pond 4	Pond 5	Pond 6
Burkholderia cepacia	Typically found in water and soil. Group of bacteria, can cause pneumonia in humans and disease in onion and tobacco plants.	A/B/C	A/B/C/D	A/B/C/D	B/D	A/B/C/D	A/C/D
Mycobacterium intracellulare	Typically found in water and soil. Can cause lung disease in humans.	C/D	B/D	A/B/C/D	A/B/C/D	A/B/C/D	A/B/C/D
Burkholderia pseudomallei	Soil-dwelling bacterium. Can infect humans, animals and plants, causing meliodosis in humans (symptoms across body but often presents as skin lesions/pneumonia).	A/B/C/D	A/B/D	A/B/C/D	A/B/C/D	A/B/C/D	A/B/C
Mycobacterium tuberculosis	Causative agent of tuberculosis in humans, which is spread via air droplets. Similar species also infect animals.	A/B/C/D	A/B/C/D	A/B/C/D	A/B/C/D	A/B/C/D	A/B/C
Pseudomonas aeruginosa	Found in most environments, including soil, water and human skin. Infects humans, animals and plants. In humans, it typically causes respiratory, urinary and skin infections.	A/C/D	A/B/D	A/B/C/D	A/B/C/D	A/B/C/D	A/B/C/D
Stenotrophomonas maltophilia	Ubiquitous in water and soil environments, and in plants. Can cause nosocomial (hospital-acquired) infections in humans, but this is not common.	A/C/D	A/B/D	A/B/C/D	A/C/D	A/B/C/D	A/B/D

Table S10: Wider Bangladesh policies and initiatives linked to AMR through our DPSIR application. List of drivers and pressures identified and links to Bangladesh Government policies and initiatives that are already in place to address these.

Driver/Pressure	Policies or initiatives already in place
Antibiotics readily available	National Drug Policy 2016<br><br>Pharmacovigilance and Adverse Drug Reaction Policy 2017<br><br>AMR National Action Plan 2017–2022 and NAP roadmap
Antimicrobials in commercial feed	Fish Feed and Animal Feed Act, 2010
Climate change	Bangladesh Climate Change Strategy and Action Plan 2009<br><br>Nationally determined contribution 2020 (Interim) by Ministry of Environment, Forest and Climate Change, Bangladesh
Economic growth	8th Five year plan 2020-2025<br><br>Making Vision 2041 a Reality: PERSPECTIVE PLAN OF BANGLADESH 2021-2041
Environmental pollution	Guideline for Assessment of Effluent Treatment Plant 2008<br><br>Bangladesh Standards and Guidelines for Sludge Management 2015 from the Department of Environment
Inadequate labelling of antibiotics	AMR National Action Plan 2017–2022 and NAP roadmap
Inappropriate antibiotic use in humans/other animals	AMR National Action Plan 2017–2022 and NAP roadmap<br><br>Draft "Guidelines on Antimicrobial Consumption (AMC) Surveillance in Bangladesh" (currently open for public consultation, to be made available from July 2022), from the Bangladesh Government's Directorate General of Drug Administration (DGDA)
Intensification	Making Vision 2041 a Reality: PERSPECTIVE PLAN OF BANGLADESH 2021-2041
Lack of affordable credit for investments	Bangladesh Investment Promotion and Financing Facility Project II 2017-22

Driver/Pressure	Policies or initiatives already in place
Lack of knowledge on appropriate usage/AMR	AMR National Action Plan 2017–2022 and NAP roadmap
Lack of technology for temp regulation, oxygen management, etc	National Aquaculture Development Strategy and Action Plan of Bangladesh 2013–2020
Lack of training/education on best management practices	Training materials are available from a number of organisations, such as Bangladesh Government's Department of Fisheries (DoF), Department of Youth Development (DYD), Ministry of Youth and Sports, USAID funded projects, and many NGOs
Poor access to disease advice and diagnostics	Fleming Fund activities to strengthen surveillance capacity
Poor access to healthcare	Number of strategies, as outlined on Bangladesh Government's Ministry of Health and Family Affairs webpage
Poor power infrastructure	World Bank's Rural Electrification and Renewable Energy Development Project-II (started in 2012)
Poor sanitation/WWT and access to clean water	Bangladesh Rural Water Supply and Sanitation Project 2012-16
Poor uptake of best management practices	Code of Conduct and Good Aquaculture Practice by Department of Fisheries<br><br>National Aquaculture Development Strategy and Action Plan of Bangladesh 2013–2020
